# Duration and determinants of delayed tuberculosis diagnosis and treatment in high-burden countries: a mixed-methods systematic review and meta-analysis

**DOI:** 10.1186/s12931-021-01841-6

**Published:** 2021-09-23

**Authors:** Alvin Kuo Jing Teo, Shweta R. Singh, Kiesha Prem, Li Yang Hsu, Siyan Yi

**Affiliations:** 1 Saw Swee Hock School of Public Health, National University of Singapore, National University Health System, Singapore, Singapore; 2 Department of Infectious Disease Epidemiology, Faculty of Epidemiology and Population Health, London School of Hygiene and Tropical Medicine, London, UK; 3 Yong Loo Lin School of Medicine, National University of Singapore and National University Health System, Singapore, Singapore; 4 KHANA Center for Population Health Research, Phnom Penh, Cambodia; 5 Center for Global Health Research, Touro University California, Vallejo, USA; 6 Saw Swee Hock School of Public Health, National University of Singapore, #10-01, 12 Science Drive 2, Singapore, 117549 Singapore

**Keywords:** Tuberculosis, Patient delay, Health system delay, Treatment delay, Total delay, High burden countries, Risk factors

## Abstract

**Background:**

Thirty countries with the highest tuberculosis (TB) burden bear 87% of the world’s TB cases. Delayed diagnosis and treatment are detrimental to TB prognosis and sustain TB transmission in the community, making TB elimination a great challenge, especially in these countries. Our objective was to elucidate the duration and determinants of delayed diagnosis and treatment of pulmonary TB in high TB-burden countries.

**Methods:**

We conducted a systematic review and meta-analysis of quantitative and qualitative studies by searching four databases for literature published between 2008 and 2018 following PRISMA guidelines. We performed a narrative synthesis of the covariates significantly associated with patient, health system, treatment, and total delays. The pooled median duration of delay and effect sizes of covariates were estimated using random-effects meta-analyses. We identified key qualitative themes using thematic analysis.

**Results:**

This review included 124 articles from 14 low- and lower-middle-income countries (LIC and LMIC) and five upper-middle-income countries (UMIC). The pooled median duration of delays (in days) were—patient delay (LIC/LMIC: 28 (95% CI 20–30); UMIC: 10 (95% CI 10–20), health system delay (LIC/LMIC: 14 (95% CI 2–28); UMIC: 4 (95% CI 2–4), and treatment delay (LIC/LMIC: 14 (95% CI 3–84); UMIC: 0 (95% CI 0–1). There was consistent evidence that being female and rural residence was associated with longer patient delay. Patient delay was also associated with other individual, interpersonal, and community risk factors such as poor TB knowledge, long chains of care-seeking through private/multiple providers, perceived stigma, financial insecurities, and poor access to healthcare. Organizational and policy factors mediated health system and treatment delays. These factors included the lack of resources and complex administrative procedures and systems at the health facilities. We identified data gaps in 11 high-burden countries.

**Conclusions:**

This review presented the duration of delays and detailed the determinants of delayed TB diagnosis and treatment in high-burden countries. The gaps identified could be addressed through tailored approaches, education, and at a higher level, through health system strengthening and provision of universal health coverage to reduce delays and improve access to TB diagnosis and care.

*PROSPERO registration*: CRD42018107237.

**Supplementary Information:**

The online version contains supplementary material available at 10.1186/s12931-021-01841-6.

## Background

In 1993, the World Health Organization (WHO) declared global tuberculosis (TB) emergency to make TB a high priority [1]. Twenty-five years on, TB remains one of the leading infectious causes of illness and death worldwide [2]. Despite that TB is both preventable and curable, and efforts such as the implementation of directly observed treatment short course and coordinated national TB programs worldwide, approximately 10 million people fell ill with TB, of which 1.5 million died from the disease in 2018 [2]. The cumulative reduction in the TB incidence rate globally between 2015 and 2018 stood at 6% [2], imposing a significant delay in reaching the end TB milestone of 20% [3] reduction by 2020. TB control and elimination are critical challenges in many countries. However, the burden is disproportionately borne by 30 countries, mostly in Asia and Africa, accounting for 87% of the world’s TB (both pan-TB and drug-resistant TB) and TB/HIV cases [2].

In 2018, nearly one-third of the people with TB were estimated to be undiagnosed globally [2]. The delay in diagnosis and treatment is detrimental to the patients’ prognosis and perpetuates TB transmission in the community [4] and thus poses a great challenge to eliminating TB. Therefore, identifying the factors that lead to delayed TB diagnosis and treatment is imperative in developing interventions to reduce TB incidence substantially. Collectively, recent systematic reviews have provided empirical evidence associating sociodemographic, clinical, health system, and economic factors with delayed diagnosis and treatment of TB in different countries and regions [5–11]. However, delays in diagnosis and treatment vary across countries with a different burden of the disease. From what we know, no systematic reviews have addressed delayed diagnosis and treatment of TB among countries bearing most of the global TB burden. There is also a lack of reviews that triangulate qualitative and quantitative findings to provide a more complete and all-inclusive view of the matter. Therefore, a mixed-methods systematic review and meta-analysis were undertaken to derive the determinants and duration of diagnosis and treatment delays of pulmonary TB in the high TB-burden countries.

## Methods

We structured this review following the Preferred Reporting Items for Systematic Reviews and Meta-Analysis (PRISMA)-statement [12]. The protocol of this systematic review has been published [13] and registered with the International Prospective Register of Systematic Reviews (PROSPERO) (Registration Number CRD42018107237).

### Inclusion and exclusion criteria

In this review, we considered all studies conducted in the WHO high TB-burden countries—Angola, Bangladesh, Brazil, Cambodia, Central African Republic, China, Congo, Democratic People’s Republic of Korea, Democratic Republic of Congo, Ethiopia, India, Indonesia, Kenya, Lesotho, Liberia, Mozambique, Myanmar, Namibia, Nigeria, Pakistan, Papua New Guinea, the Philippines, Russian Federation, Sierra Leone, South Africa, Tanzania, Thailand, Vietnam, Zambia, and Zimbabwe. We included studies that reported on individual and interpersonal risk factors, social and physical environment, health systems, and policies associated with delayed TB diagnosis and treatment initiation published between 2008 and 2018. The factors could be self-reported, ascertained by health providers, or abstracted from medical charts or programs/administrative records.

We included study populations comprising presumptive TB (persons presenting with signs and/or symptoms suggestive of TB) and people with TB (new diagnosis, previously treated, and those without a known history of previous TB treatment) regardless of HIV and bacteriological status. We included observational (cross-sectional, case–control, retrospective, and prospective cohort design) and qualitative studies published in English or Chinese. Systematic reviews, meta-analyses, scoping reviews, intervention studies, publications in the form of letters and reviews, and studies lacking and/or unclear reporting of key outcomes were excluded.

Our primary outcomes were—(1) patient delay: the time interval between the onset of symptoms and the first encounter with healthcare professionals; (2) health system delay: the time interval between the first encounter with healthcare professionals and the diagnosis of pulmonary TB; (3) treatment delay: the time interval between TB diagnosis and TB treatment initiation; and (4) total delay: the time interval between onset of symptoms and TB treatment initiation. As there were no universal cut-offs [8] to a duration that constituted delay, we treated delay in this review as how they were defined in individual studies. We did not exclude studies based on the delay thresholds defined in individual studies.

### Literature search strategy and study selection

First, we conducted a preliminary search of articles on PubMed and EMBASE to develop a set of appropriate Medical Subject Heading terms, index terms, and keywords [13], centered around three domains (population/problems: tuberculosis, outcomes: health-seeking behaviours; delays; barriers, countries: high burden countries). Using these identified search terms structured with Boolean logic operators (AND and OR), we contextualized the search strategies in PubMed, EMBASE, CINAHL, and PsycInfo (Additional file 1). The search fields included title, abstract, keywords, and text words. We also reviewed the reference list of key articles for additional studies. We managed all identified citations into EndNote X8 (Clarivate Analytics, Philadelphia, USA). Duplicates were removed, and the remainder was exported to Microsoft Excel (Microsoft Corporation, Washington, USA) for further assessment. AKJT and SRS independently screened the titles, abstracts, and full-text articles based on the inclusion and exclusion criteria. Interrater agreements for the titles and abstract screening between the reviewers were high (agreement = 98%, Cohen’s kappa = 0.95, and Krippendorf alpha = 0.95), and discrepancies were discussed. The two primary reviewers were able to resolve all the discrepancies without having to involve a third reviewer. The search and selection processes were conducted and presented in accordance with the PRISMA guidelines.

### Data extraction

Study characteristics and data on risk factors were extracted independently by two authors (AKJT and SRS). We recorded study and participants’ characteristics, exposure variables (various factors associated with delays reported by individual study), primary outcome measures, and study quality assessment scores using a standard form. Data on variables to be included in the meta-analysis were extracted by one author (AKJT) and subsequently reviewed by a second author (KP). This included duration of delay (median and interquartile range/range and mean and standard deviation) and the effect sizes (crude and adjusted odds ratios) for exposures of interest.

### Quality assessment

The quality of the selected non-randomized (quantitative) and qualitative studies was critically evaluated using the Newcastle–Ottawa Scale for cross-sectional studies, case–control studies, and cohort studies, and the Critical Appraisal Skills Program (CASP) tool, respectively [14–16]. For non-randomized quantitative studies, the assessment was made based on four main domains—(1) selection of samples (representativeness, sample size, definition and selection of cases and controls (for case–control studies), and non-response rate), (2) comparability of groups included in the analyses, (3) the ascertainment of exposures and outcomes, and (4) the statistical tests applied in the studies. A score of 1 was given to individual questions if the criterion was satisfied and 0 if the criterion was not satisfied or not justified. The highest possible score for cross-sectional studies was 10 (5 for selection, 2 for comparability, and 3 for outcomes). The highest possible score for case–control studies was 9 (4 for selection, 2 for comparability, and 3 for exposure). The highest possible score for cohort studies was 9 (4 for selection, 2 for comparability, and 3 for exposures). Studies that scored 0–3 were regarded as low quality (LQ), 4–6 were regarded as moderate quality (MQ), and ≥ 7 were regarded as high quality (HQ).

For qualitative studies, the assessment was made based on ten questions regarding the results, validity, and the value of the research. We gave a score of 1 if the paper fulfilled a criterion, 0.5 if we could not tell if the paper fulfilled a criterion, and 0 if the paper did not fulfill a criterion. A score of 0–5 equated to LQ study, a score of 6–7 equated to MQ study, and a score of ≥ 8 equated to HQ study. The final synthesized qualitative findings were graded based on the dependability and credibility of the findings using the ConQual approach [17].

### Data synthesis and analyses

We described the studies by the populations, countries, study designs, and sample sizes. Countries were grouped by WHO region and categorized as low-income economies (LIC)—gross national income (GNI) per capita $1,025 or less in 2018; lower-middle-income economies (LMIC)—GNI per capita between $1,026 and $3,995; upper-middle-income economies (UMIC)—GNI per capita between $3,996 and $12,375 according to World Bank classification in 2019 [18]. We reported the independent variables significantly associated with the patient, health system, treatment, and total delays. Results from the multivariable analyses preceded bivariate for studies that reported both bivariate and multivariable analyses.

Median and interquartile range/range for the duration of delays in days were extracted and used to estimate a pooled median, i.e., median of study-specific medians [19]. We pooled weighted medians by incorporating study-specific sample sizes [19]. For patient delay, we excluded two studies [20, 21] from China with sample sizes > 10,000 because the pooled weighted medians were heavily skewed, including only estimates of the study with the largest sample size [21].

For independent variables (risk factors), effect sizes were extracted and used to calculate pooled odds ratios (OR) and their 95% confidence interval (CI). We pooled effect sizes of covariates from studies that utilized similar delay thresholds if data were available in more than two studies and duration of delays by meta-analysis using R (R Foundation for Statistical Computing, Vienna). We pooled effect sizes for studies that defined patient delay using threshold values of 14–15 days (n = 5), 20–21 days (n = 7), 28–30 days (n = 17); and health system delay using threshold values of 14–15 days (n = 5). We found five studies that reported treatment delay using a threshold value of 7 days. However, the studies did not report similar covariates with effect sizes that allowed pooling. Where adjustments for covariates had been performed, the data from the adjusted model were pooled.

We quantified between-study heterogeneity using Chi-square statistic Q, I^2^, and Tau [22]. We estimated the pooled OR and its 95% CI using a Bayesian random-effect model for each meta-analysis, which accounted for between-study heterogeneity [23]. The estimates for Tau and I^2^ statistics were presented together with the pooled estimates and the 95% CI. We used the inverse of the effect size variance to determine the pooling weights. We assessed the association of the primary outcomes and (1) sociodemographic and economic variables: sex, urbanicity; (2) behavioral variables: smoking, alcohol use, TB knowledge; and (3) clinical and health services-related variables: hemoptysis, weight loss, fever, chest pain, night sweats in the meta-analyses.

We extracted qualitative findings and sample quotes reported in qualitative and mixed-method studies verbatim. The extracted data were annotated and analyzed using NVIVO 12 (QSR International). We retrieved references deductively and applied thematic analyses to categorize the textual references. Two authors (AKJT and SRS) coded the data independently. Discrepancies, code definitions, and the emergence of sub-themes were discussed. The results were presented by income categories that the high-burden countries represent.

## Results

### Study selection

**Fig. 1:**
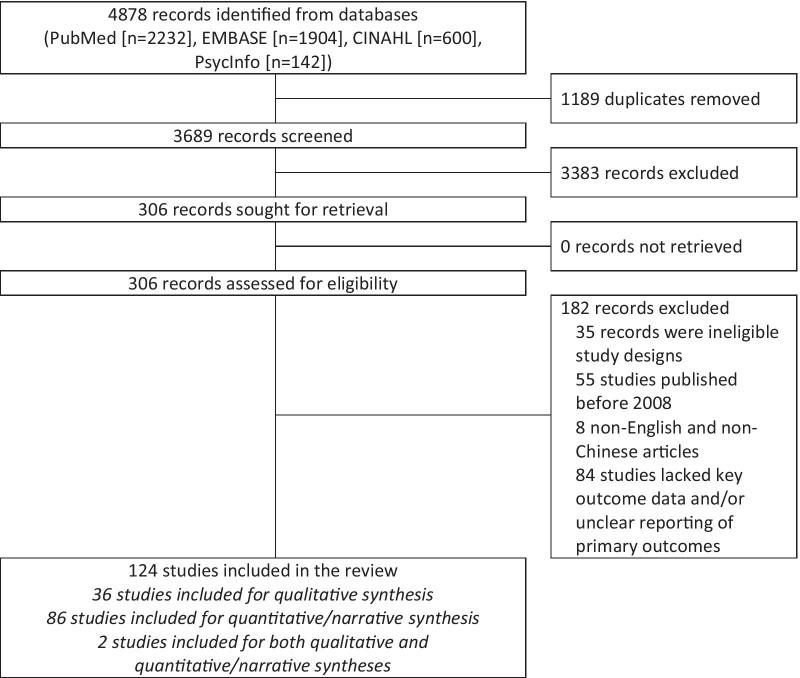
PRISMA flow diagram for identification of studies via databases

The systematic review process is presented in Fig. 1. A total of 4878 records were identified from electronic database searches. Following the removal of duplicates (n = 1189) and non-relevant records (n = 3383), 306 records were assessed for eligibility. Of these, 182 articles were further excluded. Finally, 124 articles were reviewed. A qualitative synthesis was performed for 36 studies. We conducted quantitative and narrative synthesis on 86 studies. Two mixed-method studies underwent both qualitative and quantitative/narrative synthesis. We found large heterogeneity among studies included in the meta-analyses—1 (7%) had I^2^ ≤ 50%, 14 (93%) had I^2^ > 50%, and 13 (87%) had I^2^ > 75%.

### Study characteristics and quality assessments

**Fig. 2:**
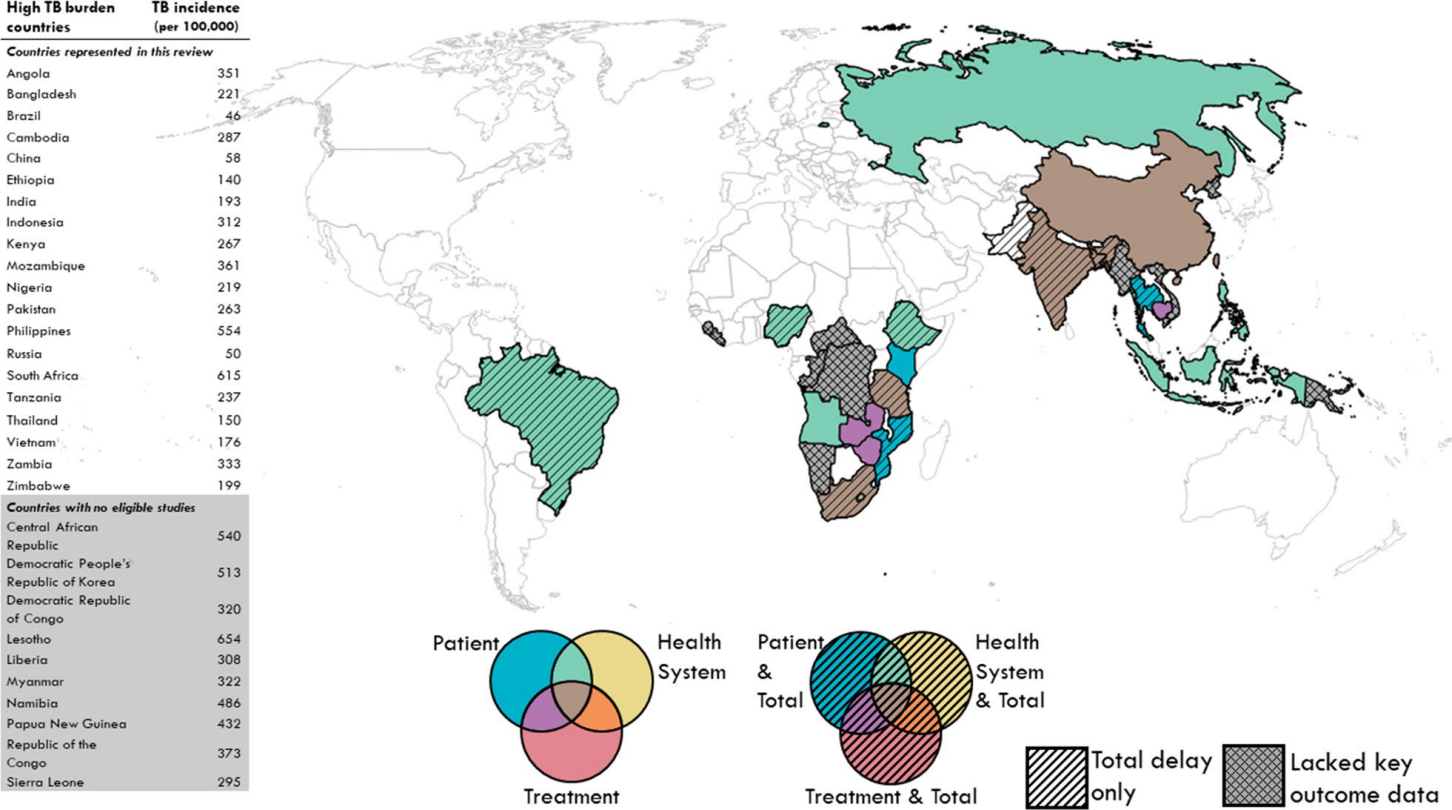
Geographical coverage of studies published between 2008 and 2018 included in this systematic review of delayed diagnosis and treatment of pulmonary tuberculosis. The 30-high tuberculosis (TB) burden countries which have been designated by the World Health Organization are outlined in black. Of them, countries with studies presenting various types of delay are categorized by the various colors. For example, countries shaded in green had studies presenting both patient and health system delay, and those with diagonal strips presented total delay too. Some of the high TB burden countries shaded in grey had no studies identified here or lacked key outcome data. The table on the left represents the TB incidence per 100,000 population of high TB burden countries in 2019. Rows shaded in grey represent countries that were not included in this review either due to data unavailability or lack of key outcome data

These studies described data from 18,759 presumptive TB and 131,142 people with TB [20, 21, 24–109], 1659 in-depth and structured interviews, and 87 focus groups [48, 96, 110–145] from 19 countries in three continents (Table 1). A total of 14 countries were classified as lower-income (LIC) and lower-middle-income economies (LMIC), and five were classified as upper-middle-income economies (UMIC) [18]. Patient delay was reported in 103 studies, health system delay in 29 studies, treatment delay in 18 studies, and total delay in 21 studies. Of the 30 high TB-burden countries, 11 countries were not included in this review, either due to data unavailability or lack of key outcome data (Fig. 2). After assessments of study quality, a total of 81 HQ studies, 40 MQ studies, and one LQ study were identified. Two mixed-methods studies scored MQ/HQ and HQ/MQ for the quantitative and qualitative components, respectively. The final synthesized qualitative findings were rated HQ (55%) and MQ (45%) using the ConQual method. Details of the assessments were illustrated in the Additional file 1. No studies were excluded based on the outcome of quality assessments; instead, the information was considered during data synthesis and interpretation.

**Table 1:** Characteristics of included observational and qualitative studies

Income group*	Country	Study population	Study design	Sample size and study		
				Newcastle–Ottawa scale score^†^		
				HQ	MQ	LQ
Patient delay						
LIC	Ethiopia	People with TB	Cross-sectional	216[24], 296[25], 360[26], 382[27], 398[28], 425[29], 605[30], 706[31], 924[32]	129[33], 201[34], 226[35]	
		People with presumptive TB	Case–control	838[36]		
			Cross-sectional	476[37], 843[38], 1006[39]	663[40], 763[41]	
	Mozambique	People with TB	Cross-sectional		622[42]	
	Tanzania	People with TB	Cross-sectional	639[43]	206[44]	
		People with presumptive TB	Cross-sectional	3388[45]		
LMIC	Angola	People with TB	Cross-sectional	385[46]		
	Bangladesh	People with TB	Cross-sectional		7280[47]	
	Cambodia	People with TB	Mixed-methods		96[48]	
	India	People with TB	Cross-sectional	216[49], 234[50]	150[51], 261[52]	
		People with TB (children)	Cross-sectional		175[53]	
		People with presumptive TB	Cross-sectional	437[54]		
	Indonesia	People with presumptive TB	Cross-sectional	194[55]	746[56]	
	Kenya	People with TB	Cross-sectional			230[57]
		People with presumptive TB	Cross-sectional		426[58]	
	Nigeria	People with TB	Cross-sectional	160[59], 450[60]	102[61]	
	Zambia	People with presumptive TB	Cross-sectional	6708[62]		
	Zimbabwe	People with TB	Cross-sectional	383[63]		
UMIC	Brazil	People with TB	Cross-sectional	139[64], 153[65]	97[66], 101[67], 199[68], 218[69], 304[70]	
		TB-HIV co-infection	Prospective cohort	201[71]		
	China	People with TB	Cross-sectional	314[72], 1126[73], 2280[74]	146[75], 259[76], 314[77], 323[78], 819[79], 1083[80]	
			Prospective cohort		202[81]	
			Retrospective cohort	4677[82], 10356[20]	75401[21]	
		People with presumptive TB	Cross-sectional		1005[83]	
	Russia	People with TB	Cross-sectional	105[84]		
	South Africa	General population	Cross-sectional		1020[85]	
		People with presumptive TB	Cross-sectional		104[86]	
		TB-HIV co-infection	Prospective cohort	891[87]		
	Thailand	People with TB	Cross-sectional	443[88]	199[89]	
Health system delay						
LIC	Ethiopia	People with TB	Cross-sectional		201[34]	
	Nigeria	People with TB	Cross-sectional	470[90]		
LMIC	Angola	People with TB	Cross-sectional	385[46]		
UMIC	Brazil	People with TB	Cross-sectional		218[69], 304[70], 305[91]	
	China	People with TB	Cross-sectional	314[72]	146[75]	
			Prospective cohort		202[81]	
			Retrospective cohort	4677[82]		
	South Africa	TB-HIV co-infection	Cross-sectional	480[92]		
Treatment delay						
LIC	Tanzania	People with TB	Cross-sectional	1161[93]		
LMIC	Bangladesh	People with TB	Cross-sectional		123[94]	
	Cambodia	People with TB	Mixed-methods		96[48]	
	India	People with TB	Cross-sectional	234[50], 344[95]	150[51]	
			Mixed-methods	2027[96]		
			Retrospective cohort	662[97], 1800[98]		
	Zimbabwe	People with TB	Retrospective cohort	2443[99]		
UMIC	China	People with TB	Cross-sectional	314[100]		
			Retrospective cohort	4677[82]		
	South Africa	People with TB	Cross-sectional	210[101]		
Total delay						
LIC	Ethiopia	People with TB	Cross-sectional	216[24], 296[25], 328[102], 382[27]	201[34]	
	Mozambique	People with TB	Cross-sectional		622[42]	
	Tanzania	People with TB	Cross-sectional		206[44]	
LMIC	Bangladesh	People with TB	Cross-sectional		7280[47]	
	India	People with TB	Cross-sectional	216[49], 289[103]		
			Retrospective cohort	656[104]		
	Indonesia	People with TB	Cross-sectional		1116[105]	
	Nigeria	People with TB	Cross-sectional	450[60]		
	Pakistan	People with TB	Cross-sectional	844[106]	252[107], 269[108]	
UMIC	Brazil	People with TB	Case–control	242[109]		
			Cross-sectional		304[70]	
	South Africa	People with TB	Cross-sectional	210[101]		
		TB-HIV co-infection	Prospective cohort	891[87]		
	Thailand	People with TB	Cross-sectional	443[88]		

Income group*	Country	Study population	Methods of analysis	Study and sample size	ConQual rating^‡^	CASP score^§^
*Qualitative studies*						
LIC	Ethiopia	People with TB, contacts of people with TB, and health care workers	Phenomenological analysis	5 IDIs and 2 FGDs [110]	HQ	HQ
		People with TB	Thematic analysis	26 IDIs [111]	HQ	HQ
		People with TB and policymakers	Thematic analysis	19 IDIs [112]	HQ	HQ
	Mozambique	Caretakers of people with TB	Content analysis	35 IDIs [113]	HQ	HQ
	Tanzania	People with TB and traditional healers	Content analysis	32 IDIs [114]	HQ	HQ
LMIC	Bangladesh	People with TB	Qualitative analysis of open-ended survey questions	229 interviews [115]	MQ	MQ
		People with TB and health care workers	Qualitative analysis using apriori codes	24 IDIs [116]	HQ	HQ
	Cambodia	People with TB, health care workers, and community volunteers	Thematic analysis	43 IDIs and 6 FGDs [48]	MQ	HQ
		People with TB and the general population		13 FGDs [117]	HQ	HQ
	India	Health care workers	Thematic analysis	16 IDIs [118]	HQ	HQ
		People with TB	Not presented in the article	76 IDIs [119], 75 structured interviews [96]	MQ	MQ
				108 structured interviews [120]	MQ	HQ
			Qualitative analysis of open-ended survey questions	229 interviews [115]	MQ	MQ
		People with TB and health care workers	Qualitative analysis using apriori codes	19 IDIs [121]	MQ	HQ
			Thematic analysis	71 IDIs [122]	HQ	HQ
	Indonesia	People with TB and community volunteers	Thematic analysis	67 IDIs and 6 FGDs [123]	HQ	HQ
		People with TB, TB survivors, village leaders, and community volunteers	Not presented in the article	50 IDIs and 3 FGDs [124]	HQ	HQ
	Nigeria	General population	Thematic analysis	56 IDIs [125]	MQ	HQ
	Philippines	People with TB and the general population	Thematic analysis	22 IDIs and 3 FGDs [126]	HQ	HQ
	Zambia	People with TB and community volunteers	Thematic analysis	30 IDIs and 6 FGDs [127]	MQ	HQ
	Zimbabwe	People with presumptive TB	Grounded theory	20 IDIs [128]	HQ	HQ
UMIC	Brazil	Health care workers	Discourse analysis	16 IDIs [129]	MQ	HQ
		People with TB	Content analysis	23 IDIs [130]	MQ	HQ
			Thematic analysis	7 IDIs [131]	MQ	HQ
			Discourse analysis	7 IDIs [132]	HQ	MQ
	China	People with TB	Qualitative analysis of open-ended survey questions	70 interviews [133]	MQ	MQ
		People with TB (migrants)	Thematic analysis	34 IDIs [134]	MQ	HQ
		People with TB (migrants), People with presumptive TB, and health care workers	Framework approach	60 IDIs and 12 FGDs [135]	MQ	HQ
		People with TB, health care workers, policymakers, and community volunteers	Thematic analysis	47 IDIs and 5 FGDs [136]	MQ	HQ
	Russia	People with TB	Grounded theory	5 FGDs [137]	HQ	HQ
		People with TB and health care workers		32 IDIs and 11 participants in FGDs (number of FGDs not specified) [138]	HQ	HQ
	South Africa	Contacts of people with TB, health care workers, policymakers, and people with TB (miners)	Thematic analysis and grounded theory	104 applied ethnography using formal/informal IDIs, FGDs, field notes, and participant observations [139]	HQ	HQ
		Health care workers, village leaders, and researchers	Thematic analysis	12 IDIs [140]	HQ	HQ
		People with TB		41 IDIs [141]	MQ	HQ
		People with TB, contacts of people with TB, and health care workers		25 IDIs and 4 FGDs [142]	HQ	HQ
		People with TB, the general population, and community volunteers		93 reports from participatory research and participants observation [143]	HQ	HQ
		People with TB and general population	Thematic analysis and grounded theory	8 IDIs [144]	HQ	HQ
	Thailand	People with TB (migrants) and health care workers	Thematic analysis	12 IDIs and 11 FGDs [145]	MQ	HQ

Each number at the normal line of type in each cell referred to the sample size of each discrete study that shared the respective characteristics (country, study population, study design, and study quality). The number/s in bracket indicate the source article/s. Blank cells indicated that no studies of a particular set of characteristics were identified and included in this review

*CASP* critical appraisal skills program; *FGD* focus group discussions; *HQ* high quality, *IDI* in-depth interviews; *LIC* low-income countries, *LMIC* lower-middle-income countries, *LQ* low quality; *MQ* moderate quality, *TB* tuberculosis, *UMIC* upper-middle-income countries,

*Based on World Bank classification. Low-income economies—gross national income (GNI) per capita $1,025 or less in 2018; lower-middle-income economies—GNI per capita between $1,026 and $3,995; upper-middle-income economies—GNI per capita between $3,996 and $12,375

^†^Study quality was assessed using the Newcastle–Ottawa scale. The highest possible score for cross-sectional studies was 10 (5 for selection, 2 for comparability, and 3 for outcome). The highest possible score for case–control studies was 9 (4 for selection, 2 for comparability, and 3 for exposure). The highest possible score for cohort studies was 9 (4 for selection, 2 for comparability, and 3 for exposure). Studies that scored 0–3 were regarded as LQ, 4–6 were regarded as MQ, and ≥ 7 were regarded as HQ

^‡^All papers were pre-ranked (high, moderate, low), and the levels were adjusted according to the dependability and credibility of the findings. We pre-ranked all papers as high. The ranking remained high if the papers were regarded as dependable, and the findings were unequivocal. We downgraded the paper from high to moderate if the papers scored 3 or less in terms of dependability or scored a mix of unequivocal and credible in terms of credibility

^§^CASP for qualitative study had 10 questions to appraise the paper critically. We gave a score of 1 if the paper fulfilled a criterion, 0.5 if we could not tell if the paper fulfilled a criterion, and 0 if it did not fulfil a criterion. A score of 0–5 equated to LQ study, a score of 6–7 equated to MQ study, and a score of ≥ 8 equated to HQ study

### Patient delay

**Fig. 3:**
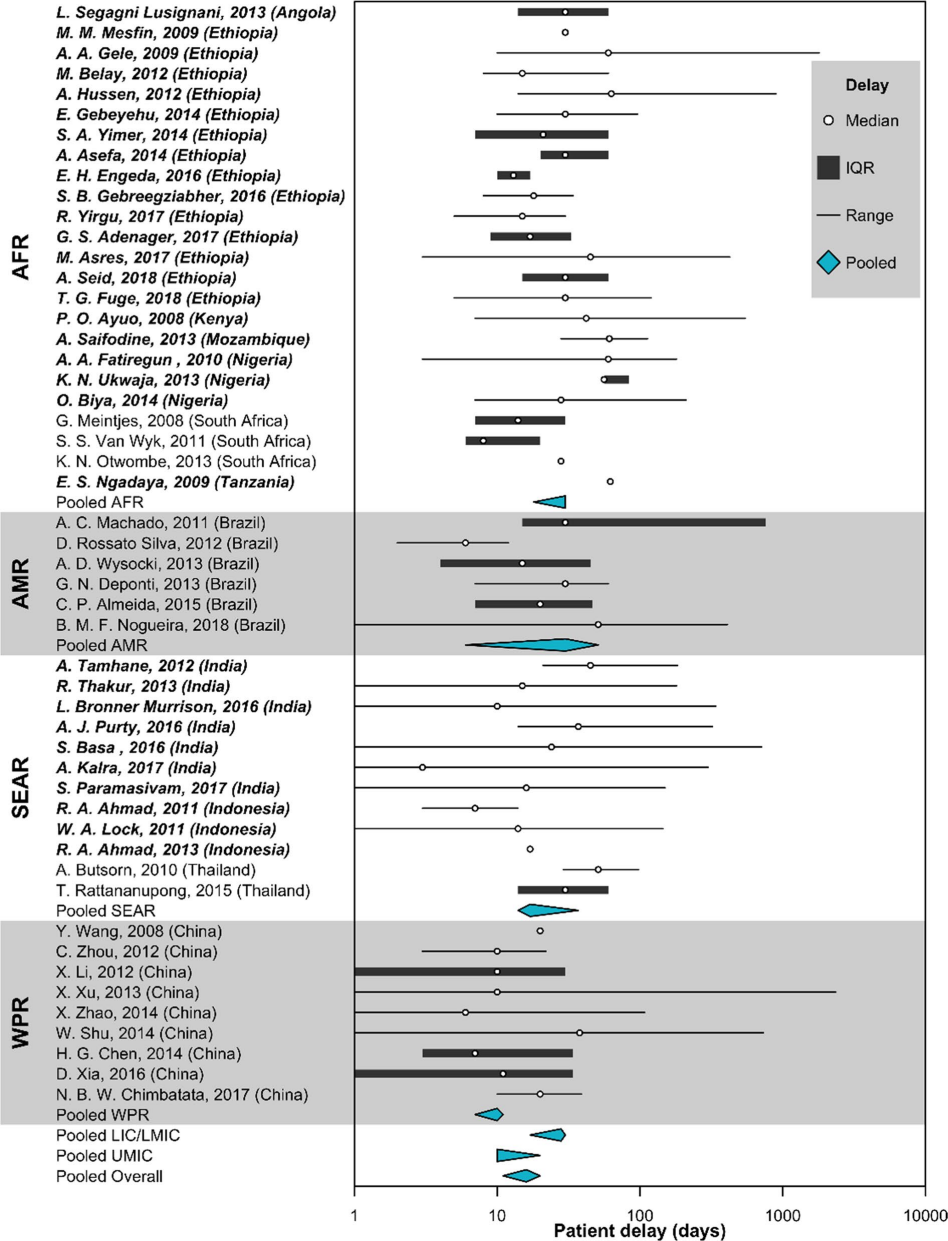
Duration of patient delay by regions reported in high tuberculosis burden countries. Countries were grouped by WHO regions (*AFR* African region, *AMR* Region of the Americas, *SEAR* South-East Asia region, *WPR* Western Pacific region). Countries were also categorized as (i) LIC (low-income countries), (ii) LMIC (low-middle income countries), or (ii) UMIC (upper-middle income countries) as designated by the World Bank in 2019. Studies from LIC and LMIC were in bold. Patient delay (in blue) was pooled by the countries’ economic status using Weighted Medians of Medians methods by McGrath (2019). The estimates were weighted by sample sizes of the studies. Pooled results for LIC and LMIC were not presented separately due to insufficient studies from LIC. Duration of delay in days were presented in the log scale

**Fig. 4:**
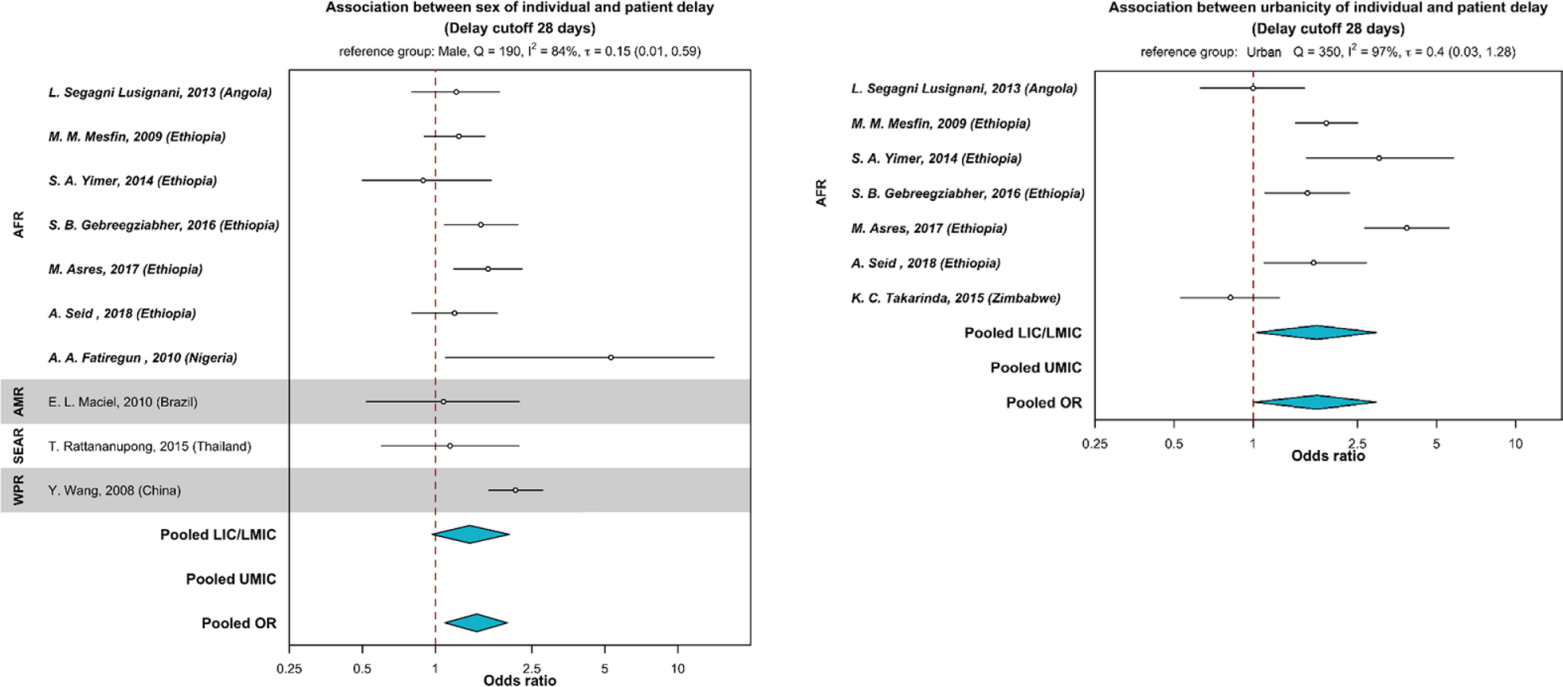
Association between sex of individuals, urbanicity and patient delay. Countries were grouped by WHO region (*AFR* African region, *AMR* Region of the Americas, *SEAR* South-East Asia region, *WPR* Western Pacific region) and categorized as (i) LIC/LMIC (low- or lower-middle-income countries), or (ii) UMIC (upper-middle-income countries) as designated by the World Bank in 2019. The reference group for sex (left panel) was male and urbanicity (right panel) was urban. The odds ratio (OR) were pooled (in blue) by countries’ economic status using Bayesian random-effects meta-analysis. Odds ratios are presented in the log scale

**Fig. 5:**
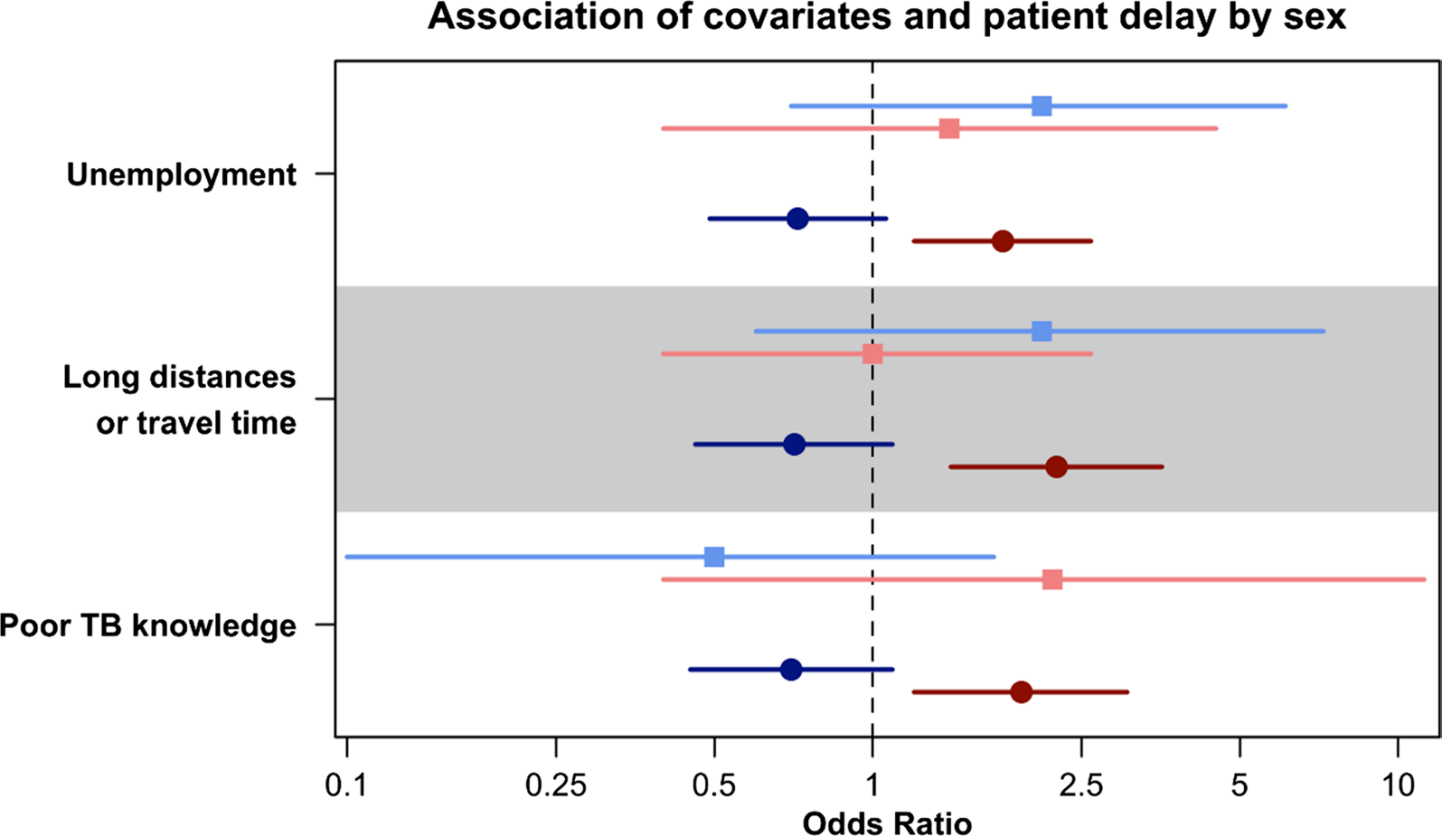
Subgroup analysis of patient delay and selected covariates by sex of the individual. Tamhane et al. (2012), represented as square points, and Mfinanga et al. (2008), represented as round points, provided sex-specific association of patient delay and three covariates; i.e., being unemployed, having to travel long distances or long travelling time, and having poor TB knowledge. The sex-specific odds ratio, in the log scale, for males are presented in hues of blues and for females in hues of reds

The pooled median patient delay (Fig. 3) in LIC and LMIC was 28 days (95% CI 17–30). The pooled median patient delay in UMIC was 10 days (95% CI 10–20). The overall median patient delay in high TB burden countries was 16 days (95% CI 11–20). In the meta-analysis and narrative synthesis of quantitative data (Table 2), females (pooled OR 1.48. 95% CI 1.09–1.98, P = 0.01) were more likely to delay care-seeking for TB (Fig. 4). Qualitative studies highlighted limitations for women to seek healthcare [115, 117, 125, 126, 130]. Women reported economic constraints and power imbalances in the decision-making process as barriers to care-seeking [115, 117, 125, 126]. We further stratified the analysis by sex. We found that women were disproportionately affected by risk factors for patient delay (Fig. 5), such as unemployment, poor TB knowledge, and difficulties traveling a long distance to visit health facilities [43, 51]. Long-distance to health facilities was also reported by qualitative studies as a barrier to care-seeking [48, 111, 112, 114, 117, 126, 131, 136, 137, 140, 142, 145]. In addition to physical barriers, financial insecurities and economic challenges also compounded patient delay [20, 25, 28, 33, 38–40, 43, 50, 51, 66, 67, 69, 72, 76, 77, 83, 87, 88]. Among qualitative studies (Table 3), seven articles reflected on participant’s experiences where competing priorities of livelihoods and commitment to work and family led to individual care-seeking delay [48, 125–127, 134, 138, 145]. We also found that being rural residents in LIC and LMIC (Table 2 and Fig. 4) was associated with patient delay (pooled OR 1.75, 95% CI 1.01–2.94, P = 0.02). No studies from the UMIC were included in the meta-analysis for urbanicity. This review also reported other sociodemographic and economic risk factors for patient delay, such as lower education level and being older, unmarried, and unemployed. High indirect medical costs [48, 126], absence of health insurance, productivity, time, and income loss [48, 125, 127, 134, 138, 145] resulting from disease suffering further worsen household vulnerabilities and contribute to a delay in TB diagnosis and poor health outcomes (Table 2) [146].

**Table 2:** Summary of risk factors for patient delay, health system delay, and treatment delay in high TB burden countries

Risk factors	Number of studies that reported the risk factors associated with the different types of delay by economies							
	Patient delay		Health system delay		Treatment delay		Total delay	
	LIC and LMIC	UMIC	LIC and LMIC	UMIC	LIC and LMIC	UMIC	LIC and LMIC	UMIC
*Socio-demographic and economic*								
Sex								
Female	3[38, 39, 61]	4[21, 69, 82, 83]		1[69]			1[108]	
Male	3[56, 58, 62]	1[86]					3[60, 102, 107]	
Age								
Older age	5[27, 30, 31, 40, 61]	3[20, 21, 74]		1[92]	3[48, 93, 95]		5[34, 47, 60, 107, 108]	
Younger age				1[72]	1[94]			
Low education	8[26, 32, 33, 40, 46, 54, 58, 60]	2[67, 71]					1[102]	
Residence								
Rural	7[25, 26, 32–34, 47, 54]	1[21]					5[25, 34, 47, 105, 108]	1[87]
Urban	3[28, 60, 62]	1[89]					1[60]	
Sub-urban			1[46]				[106]	
In areas without health centres	1[97]							
Marital status								
Married					1[94]			
Widowed/divorced/separated/not married	1[57]	2[71, 79]			1[48]			
Finances								
Low income	5[25, 28, 33, 38, 50]	5[20, 66, 72, 77, 83]	1[90]			1[100]	2[25, 108]	
High income	2[41, 62]						1[104]	
More working days per week		1[72, 77, 78]		1[72]				
Unemployed	3[39, 40, 43]	3[67, 69, 87]					1[103]	1[87]
No health insurance		[72, 76, 77, 83]						
Cost of treatment/transport to health facilities	1[51]	1[88]						
Cost of health care incurred before diagnosis					1[50]		2[103, 106]	
Long distance/traveling time to health facilities	9[29, 33, 35, 36, 43, 52, 53, 55, 57]	4[20, 72, 77, 101]		1[72]	1[48]		1[60]	
Larger family size	3[27, 38, 49]						1[27]	
*Behavioural*								
Tobacco and substance use								
Smoking	1[41]			1[81]				
Non-smoking								1[101]
Alcohol use		1[87]					1[104]	1[87]
Recreational drug use		1[65]						1[109]
Poor TB knowledge	12[30–32, 35, 42–44, 50–52, 57, 59]	5[68, 74, 79, 83, 84]				1[100]	2[50, 106]	
Poor perceived benefit that TB is incurable	1[28]							
Poor perceived severity (perceived well and not sick)	1[40]							
Stigma	3[29, 44, 61]	2[76, 85]					1[102]	
*Health-seeking*								
Types of facilities and providers								
Traditional/spiritual medicine	3[32, 42, 44]				1[93]			
Self-medication	5[24, 27, 31, 50, 63]	1[83]					1[27]	
Private health practitioner	1[32]		1[34]		1[50]		1[50]	
Pharmacy	2[32, 58]							
Non-formal health provider	5[24, 25, 30, 31, 33]						2[50, 103]	
Rural primary health facility/non-DOTS facility	2[46, 63]		1[46]				1[106]	1[101]
Non-hospital/lower-level facilities			2[34, 90]	2[72, 75]			1[24]	
Care-seeking								
Multiple care-seeking prior to diagnosis	1[59]				2[50, 51]		5[25, 27, 50, 103, 106]	
Did not seek treatment because of first symptoms		1[64]						
Not aware of other TB patients around		1[80]						
*Clinical/health services*								
Signs and symptoms								
No chest pain	1[44]							
Cough		6[69, 70, 74, 80, 86, 88]						
Chest pain		2[70, 88]						1[109]
Cough without sputum		1[88]						
Night sweats		1[74]						
Fever		1[88]						
No cough				1[70]				
No haemoptysis		3[74, 78, 80]						
No weight loss		1[64]						
Pulmonary cavities		1[73]						
No cavitary lesion				1[70]				
Shorter duration of symptoms	1[39]							
Presence of more than 1 symptom	1[45]							
Longer duration of suspicious symptoms		1[81]						
Mild symptoms at onset		1[72]						
Co-morbidities/infection								
Presence of other known medical conditions	1[42]							
Hyperglycaemia		2[73, 74]						
HIV status not known					1[96]			
HIV negative	1[25]						1[25]	
TB-HIV co-infection/HIV positive			2[34, 90]		1[98]			2[87, 101]
Not on ART				1[92]				
High HIV viral load				1[92]				
History and types of TB								
No history of TB	4[36, 37, 39, 40]	2[71, 82]			1[99]		1[103]	
History of TB				1[82]				
Smear positive	1[32]	2[73, 82]				2[82, 101]		
Smear negative	1[27]			3[81, 82, 91]	2[48, 97]		1[27]	1[109]
Extrapulmonary TB	2[27, 34]			1[91]			3[24, 27, 34]	
Retreatment cases					5[94–98]			
Health services								
Long waiting time in the health facility			1[46]					
Untraceable contact details (loss to follow-up post diagnosis)					1[95]			
Absence of TB diagnostic services in the local health facility					1[96]			

The number at the normal line of type in each cell referred to the number of study/ies that reported the risk factors associated with the type of delay, respectively. The number/s in bracket indicate the source article/s. Blank cells indicate that no studies reported the respective risk factor. The studies are further grouped by economies based on World Bank classifications

*ART* antiretroviral therapy; *DOTS* directly observed treatment, short course; *HIV* human immunodeficiency virus; *LIC* low-income countries; *LMIC* low-middle-income countries; *NTP* national TB program; *TB* tuberculosis; *UMIC* upper-middle-income countries

**Table 3:** Emerged themes and synthesized findings from qualitative studies

Themes	Countries	Quotes
*Patient delay*		
Perceived stigma and discrimination at the workplace, within the family and the community against women, and associating TB with HIV deterred people with presumptive TB from seeking TB diagnosis and care	Bangladesh [115, 116], Brazil [129, 130], Cambodia [117], China [135, 136], Ethiopia [110], India [115, 121], Nigeria [125], Philippines [126], Russia [137], South Africa [139–144], Tanzania [114], Zambia [127], Zimbabwe [128]	“When someone says, ‘I have TB’ others will say that the person has three words [HIV].’’ [143]
		“The person would be scared that she would lose her job and that people and friends would avoid her.” [126]
Long distance to health facilities and language barrier led to delay in care-seeking and TB diagnosis	Brazil [131], Cambodia [48, 117], China [136], Ethiopia [111, 112], Philippines [126], Russia [137], South Africa [140, 142], Tanzania [114], Thailand [145]	“Well, I didn’t come to the health centre early because it is far from my village.” [111]
		“I don't understand the language, so I don't know what to do next after I finished the 15 days medication. The problem for me is the language because I can't speak Thai.” [145]
Long chains of care-seeking through multiple providers and the lack of trust in the health care system providing TB care led to delay in care-seeking and TB diagnosis	Bangladesh [115, 116], Brazil [131], Cambodia [117], China [134–136], Ethiopia [110–112], India [115, 118–122], Indonesia [123, 124], Mozambique [113], Nigeria [125], Philippines [126], Russia [137, 138], South Africa [139, 140, 142–144], Tanzania [114], Zambia [127], Zimbabwe [128]	“Government doctor did not show any interest, neither he responded to my questions. They never spoke to me at all. We went there 1–3 days and became fed-up. Even the 4th day, they did not say anything. They asked me to go here and there. It was really a horrible experience to run around there. So, finally, we decided and went to private” [122]
		“We usually try many other methods first, and the hospital is the last choice.” [117]
Gender-specific factors such as men dominating and owning the decision-making power in the family, more economic constraints for women to seek healthcare, and men concealing health issues or denying disease severity by substance (alcohol and nicotine) abuse led to delay in care-seeking and TB diagnosis	Bangladesh [115, 116], India [115], Nigeria [125], Russia [137, 138], South Africa [144], Thailand [145], Zimbabwe [128] (alcohol and nicotine) abuse led to delay in care-seeking and TB diagnosis	“There are very few women in my community who can afford the costs of transportation to the hospital and to pay the hospital fees.” [125]
		“My husband told me to go to my parent’s home. He refused to give me money for the cost of treatment. My neighbour did not help me that much either.” [115]
		“When I drink, nothing is bad for me! Illness flies out with alcohol. You don’t feel it. Alcohol softens everything, all diseases. When you drink, you do not pay attention to illness. Well, today you sneeze, cough, but it will pass! In the morning, you wake up, something squeaks, whistles; you groan but go anyway, then you forget about it during work.” [138]
Competing priorities of livelihood, work, and family led to delay in care-seeking and TB diagnosis	Cambodia [48], China [134], Nigeria [125], Philippines [126], Russia [138], Thailand [145], Zambia [127]	“They had to earn money for their families and had no time for illness and examinations.” [138]
		“I work almost every day except on market days on the farm and Sundays or if there are special occasions. I usually return in the late afternoon to cook for my husband and children. So, if I should go to the hospital in the morning hours as I am told that is when they open and return in the afternoon, that whole day is gone.” [125]
Poor knowledge regarding TB symptoms and treatment and the availability of free treatment policy were barriers to early healthcare-seeking	Brazil [129], China [134, 135], Ethiopia [110, 112], India [121], Indonesia [123], Philippines [126], Russia [138], South Africa [140, 142], Tanzania [114], Zambia [127], Zimbabwe [128]	“TB is not yet a disease that people recognise, then any respiratory problem is associated with virus diseases, flu, smoke, the dust of the street, all but a disease like TB.” [129]
		“Some participants in rural areas were sceptical whether the free treatment actually existed” [123]
People with presumptive TB delayed care-seeking due to low perceived severity of symptoms, low perceived susceptibility to TB, believed that TB is hereditary or retribution for sinful behaviour, blame others for the delay and then overpowered by hopelessness	Bangladesh [115], Cambodia [48, 117], Ethiopia [110], India [115, 119], Indonesia [124], Mozambique [113], Philippines [126], Russia [137], Tanzania [114]	“They also don’t take the symptoms seriously, they just assume that is a flu.” [142]
		“My uncle suspected that she [respondent’s mother] had TB. But my mother said that in her family, no one had ever had TB … She still rejected the idea and insisted that no one in her family ever had this TB. She asked us to stop referring her [to get medical help]” [117]
		“For all patients that had initially thought they were cursed, the fact that their disease was diagnosed in the hospital was perceived as a proof that TB is a punishment of God rather than caused by witchcraft forces. Now I realise it has nothing to do with witchcraft, like I thought in the past. It is a punishment of God. He pushes the wind that contains TB in the direction of the person that will subsequently develop TB.” [114]
		“I had been sick for two years. I went to a traditional healer, but he suggested to me to go to the hospital. Before that, my friend who also had got TB suggested to me to check my sputum because he thought I might have got TB as well. I insisted that it was not TB because I have never lived with TB patients… I have heard about TB, but I did not think I would get TB.” [124]
		“Patients referred to impoverished living conditions, unclean water and insufficient food as the reasons for their TB. These causes were linked to a sense of hopelessness and an inability to improve the conditions of one’s life.” [115]
*Health system delay*		
Poor practice at the health facilities and ignorance of TB led to a delay in TB diagnosis	Bangladesh [116], Brazil [129, 132], China [133, 135], Ethiopia [110, 111], India [118, 122], Indonesia [123, 124], Tanzania [114]	“He had told me to take injections daily, and I was taking it as advised. But he did not tell me anything. He kept on saying it is typhoid. We told him that sputum is coming while coughing. But he said it will happen like this even for typhoid also.” [122]
		“There was poor adherence of the doctors to the recommended algorithm for investigating a patient suspected to have TB.” [118]
Complicated procedures at the health facilities to reach TB diagnosis	Brazil [130, 132], China [133], India [96, 119], Philippines [126], South Africa [144]	“Referrals from the public day or tertiary hospitals to clinics were not managed smoothly and receiving clinics frequently seemed to question the referral and/or diagnosis, sending the patient away without treatment or referring the patient on to another service provider.” [144]
Lack of resources and materials in the health facilities led to a delay in TB diagnosis	Brazil [129, 130, 132], India [96], Russia [138], South Africa [144]	“Very often, we do not have enough doctors or nurses in medical sites. Our doctor is absent very often. Then we go to another one. But she might not know my situation and prescribes something at random.” [138]
		“Where the patient was diagnosed at a centre without microscopy or had to be referred for treatment to a local DOTS centre, the delay was more likely to occur.” [96]
*Treatment delay*		
Self-perception of health and unconvinced of the diagnosis and the effectiveness of TB treatment led to a delay in TB treatment initiation	Cambodia [48], India [96, 119], South Africa [141]	“Sometimes, they argued and denied (their condition). They would say they are healthy. Why did we think they had the disease? They didn't trust us because they were still feeling strong.” [48]
		“Some people distrust that the medication would not be effective. Some commented on such cases where the treatment did not work or where people repeatedly kept getting TB.” [141]
Diagnosis and treatment initiated in different facilities caused a delay in TB treatment initiation	India [119]	“Active referrals by the diagnosing provider to another provider for treatment initiation constituted the major reason for delay.” [119]
Geographical distance to health facilities and other competing priorities delayed TB treatment initiation	South Africa [141]	“Sometimes, respondents were referred for treatment, but because of work-related issues, particularly work hours and the place of work, they could not access care.” [141]
Health system factors such as lack of organization at the facilities to manage patients, poor staff attitude, and logistic issues caused a delay in TB treatment initiation	India [96], South Africa [141]	“A lack of organisation at the facilities causing delays in service and queues.” [141]
		“Complaints included that staff sent a patient away when they assumed that he was not taking treatment, treat patients like children and in a derogatory manner, blame patients for problems for which they are not responsible and for shouting and swearing at patients. These raised anger and irritation at the services. While the fear may motivate patients to remain on treatment, it can make it difficult for them to return if they did not initiate treatment.” [141]
		“Delay in the transport of drugs from the Peripheral Health Institution to the DOTS centres where the patient is supposed to start his DOTS.” [96]
Women experienced stigma due to TB diagnosis resulting in the concealment of diagnosis or being isolated	Zambia [127]	“A TB patient described that she was sent away and that in rural areas, TB treatment was not easily accessible or available. This contributed to her treatment disruption and aggravation of TB.” [127]

*DOTS* directly observed treatment, short course; *HIV* human immunodeficiency virus; *TB* tuberculosis

**Fig. 6:**
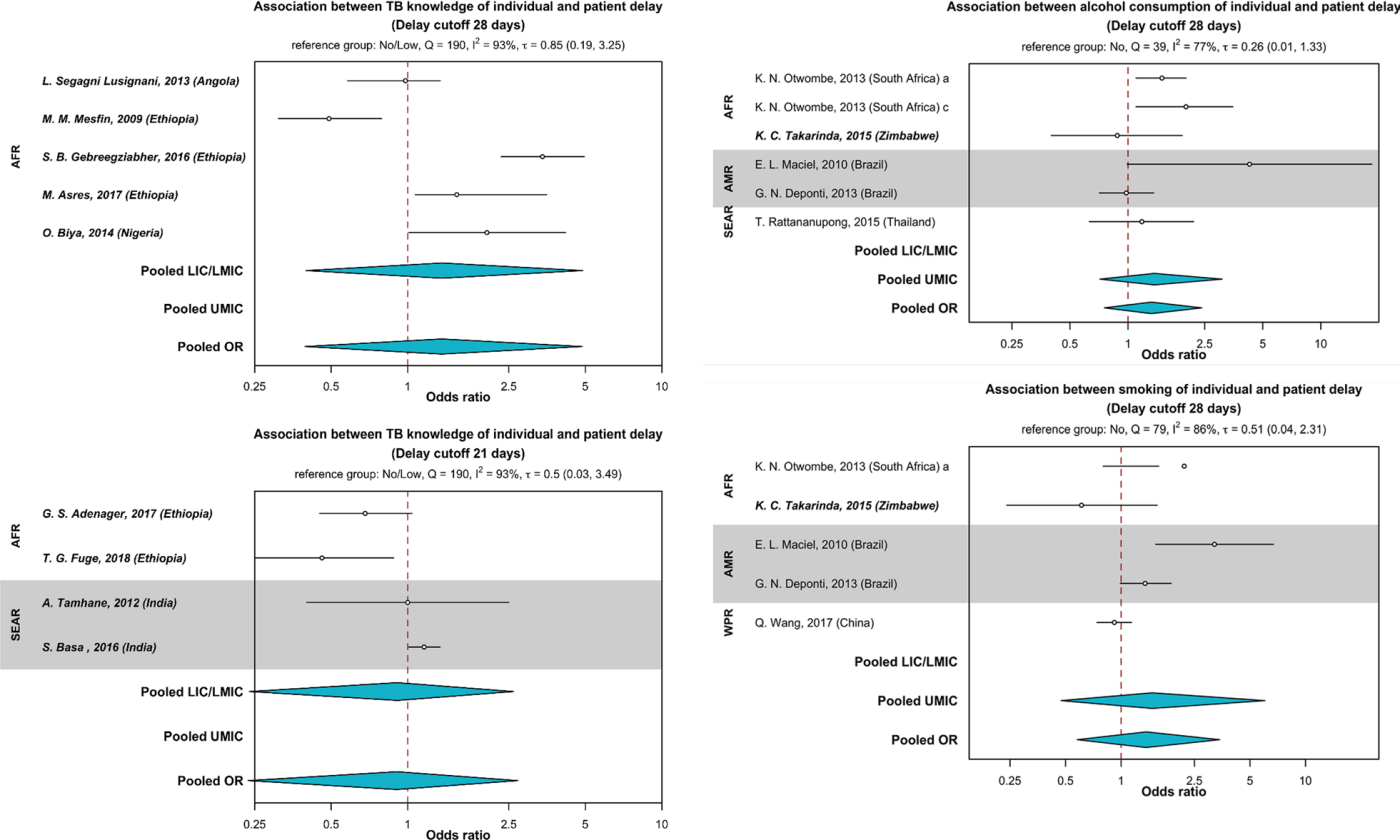
Association between TB knowledge, smoking, alcohol use and patient delay. Countries were grouped by WHO region (*AFR* African region, *AMR* Region of the Americas, *SEAR* South-East Asian region, *WPR* Western Pacific region) and categorized as (i) LIC/LMIC (low- or lower-middle-income countries), or (ii) UMIC (upper-middle-income countries) as designated by the World Bank in 2019. For TB knowledge, the top left panel pooled estimates from studies that defined patient delay threshold as 28 days. The bottom left panel pooled estimates from studies that defined patient delay threshold as 21 days. The reference group was TB knowledge (no or low). The top right panel represented pooled estimates for the association between alcohol use and patient delay. The bottom right panel represented pooled estimates for the association between smoking and patient delay. Both plots pooled estimates that defined patient delay threshold as 28 days. The reference groups were no smoking and no alcohol use, respectively. The odds ratio (OR) were pooled (in blue) by countries’ economic status using Bayesian random-effects meta-analysis. Odds ratios are presented in the log scale

Furthermore, poor TB knowledge (Table 2), unawareness of free TB treatment policies [30], low perceived susceptibility, and severity of TB was associated with patient delay [40, 48, 110, 113–115, 117, 119, 124, 126, 137]. However, the pooled estimates for TB knowledge (delay thresholds 21 days—(pooled OR 0.91, 95% CI 0.24–2.71, P = 0.62) and 28 days—(pooled OR 1.36, 95% CI 0.39–4.83, P = 0.25) were not significantly associated with patient delay in the meta-analysis (Fig. 6).

Perceived stigma and discrimination (Table 3) at the workplace, within the family, and community and associating TB with HIV deterred presumptive TB patients from care-seeking [110, 114–117, 121, 125–130, 135–137, 139–144]. From the qualitative data, we found studies that explained the use of alcohol and smoking to conceal health issues, especially among men, which resulted in delayed care-seeking [116, 128, 137, 138, 144, 145]. However, these lifestyle behaviors were not statistically significant in the meta-analysis, where the estimates from both sexes were pooled (Fig. 6). Several studies in Africa [110, 113, 114] highlighted superstitious beliefs that TB is caused by divine retributions of past misdeeds, sinful behaviors, and curses; thus, help is first sought from traditional or spiritual healers instead of a health provider. Besides, studies in Asia reported the misconception that TB is hereditary [117, 126].

**Fig. 7:**
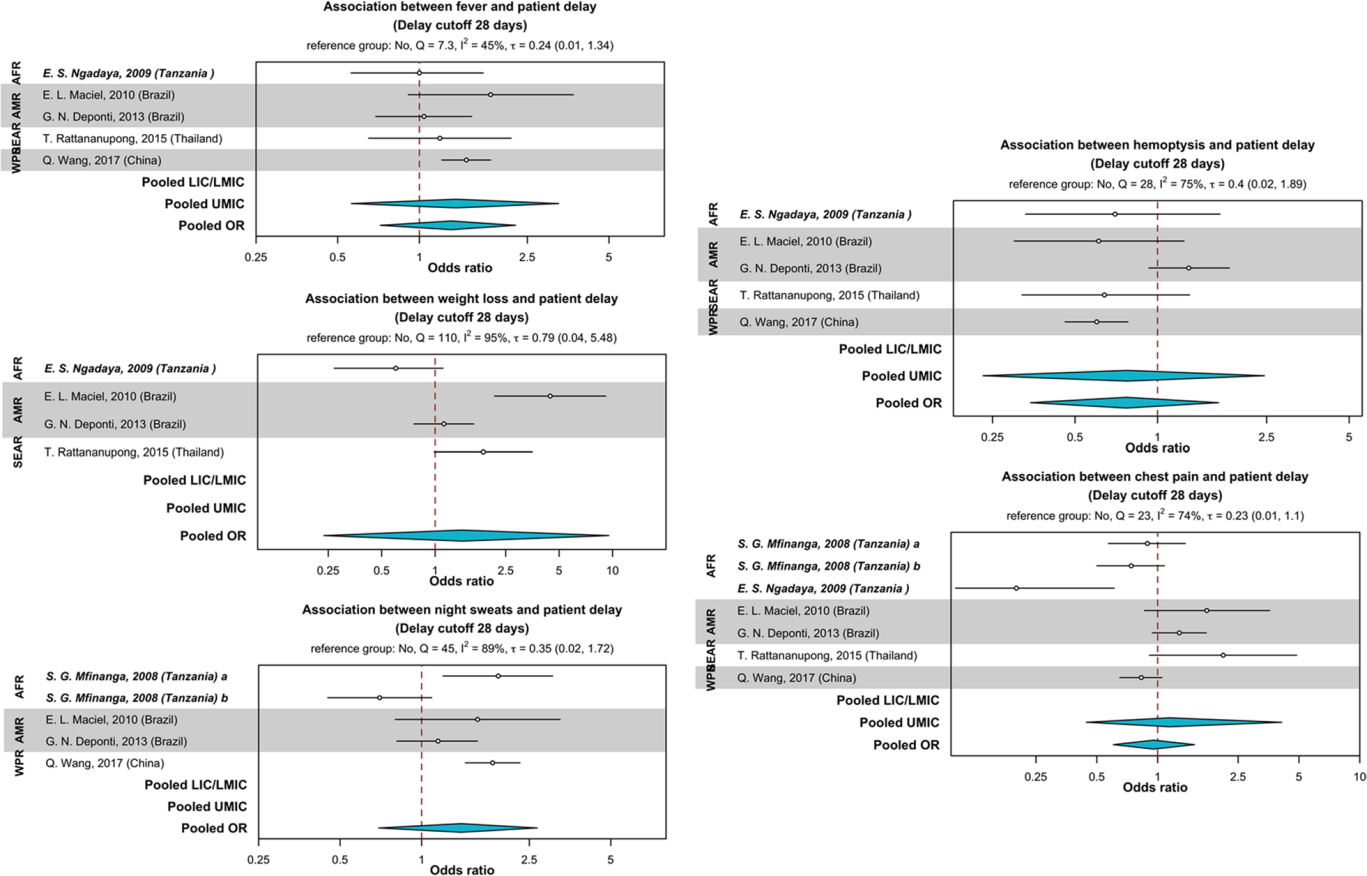
Association between TB symptoms and patient delay. Countries were grouped by WHO region (*AFR* African region, *AMR* Region of the Americas, *SEAR* South-East Asian region, *WPR* Western Pacific region) and categorized as (i) LIC/LMIC (low- or lower-middle-income countries), or (ii) UMIC (upper-middle-income countries) as designated by the World Bank in 2019. The reference group was no symptom. The patient delay threshold was 28 days. The odds ratio (OR) were pooled (in blue) by countries’ economic status using Bayesian random-effects meta-analysis. Odds ratios are presented in the log scale

Long chains of care-seeking through multiple non-formal or private health providers were also reported as a determinant of patient delay [110–128, 134, 135, 137, 138, 140, 142–144]. Qualitative data also suggested that the lack of trust in the public health care system perpetuated delays in care-seeking [122, 131, 136–139, 143, 144]. The inability of people with TB to recognize symptoms such as fever and cough that were not ascribed to TB intrinsically led to self-medication and treatment or waiting for symptoms to self-resolve due to low perceived disease severity [110, 119, 126, 128, 129, 134, 137, 140, 142]. Studies reported that the presence of cough [69, 70, 74, 80, 86, 88] was associated with patient delay compared to hemoptysis and weight loss that were perceived to be more severe (Table 2). However, the relationships between TB symptoms and patient delay were not statistically significant in the meta-analysis (Fig. 7).

### Health system delay

**Fig. 8:**
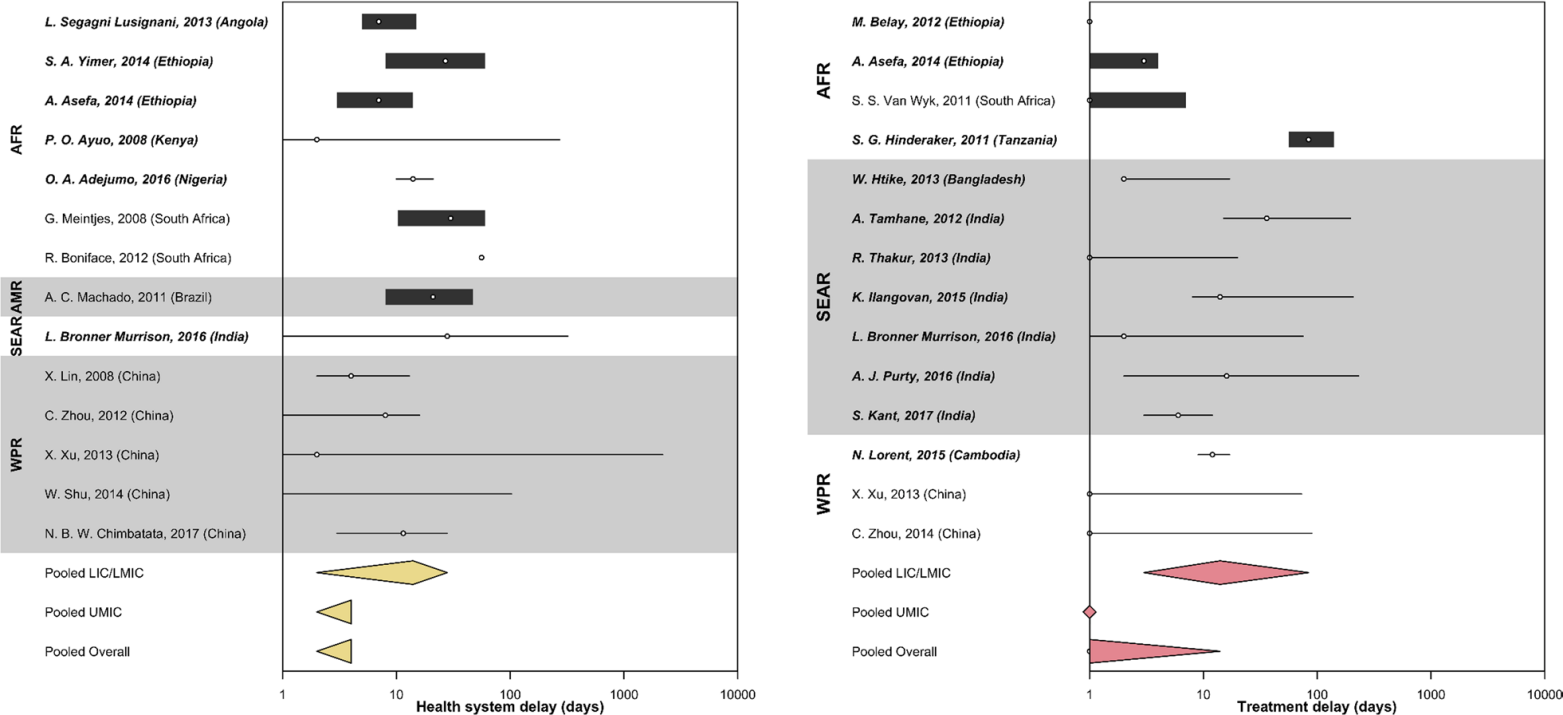
Duration of health system and treatment delay by regions reported in high tuberculosis burden countries. Countries were grouped by WHO regions (*AFR* African region, *AMR* Region of the Americas, *SEAR* South-East Asian region, *WPR* Western Pacific region). Countries were also categorized as (i) LIC (low-income countries), (ii) LMIC (low-middle income countries), or (ii) UMIC (upper-middle income countries) as designated by the World Bank in 2019. Studies from LIC and LMIC were in bold. Health system delay (in yellow) and treatment delay (in red) were pooled by the countries’ economic status using Weighted Medians of Medians methods by McGrath (2019). The estimates were weighted by sample sizes of the studies. Pooled results for LIC and LMIC were not presented separately due to insufficient studies from LIC. Duration of delay in days were presented in the log scale

The pooled median health system delay (Fig. 8) in LIC and LMIC was 14 days (95% CI 2–28). The pooled median health system delay in UMIC was 4 days (95% CI 2–4). The overall median health system delay in high TB-burden countries was 4 days (95% CI 2–4). We explored the association between sex and health system delay, and we did not find a significant relationship (Additional file 1). Twelve qualitative studies reported that poor practices and ignorance of TB among health providers at health facilities led to a delay in TB diagnosis [110, 111, 114, 116, 118, 122–124, 129, 132, 133, 135]. Seven qualitative studies explained that complicated administrative procedures at the health facilities [96, 119, 126, 130, 132, 133, 144], which could have resulted in longer waiting time [46], and complex referral system[144] that eventually prolonged health system delay. This review identified studies reporting that health system delays were associated with visiting lower-level facilities that did not provide TB services [34, 46, 72, 75, 90]. Six qualitative studies mentioned that inadequate resources and supplies in health facilities could have delayed TB diagnosis [96, 129, 130, 132, 138, 144]. Three quantitative studies reported that people with smear-negative TB were more likely to experience health system delay [81, 82, 91].

### Treatment delay

The pooled median treatment delay (Fig. 8) in LIC and LMIC was 14 days (95% CI 3–84). The pooled median treatment delay in UMIC was 0 days (95% CI 0–1). The overall median treatment delay in high TB burden countries was 1 day (95% CI 0–14). One qualitative study noted that the geographical distance to health facilities, especially when treatment was initiated in separate institutions, delayed TB treatment initiation [141]. This could be exacerbated by residing in areas without health centers nearby [97]. Health system factors such as logistical issues in drug transportation [96, 141] and the absence of TB diagnostic services in local health facilities [96, 119] compounded delay in treatment initiation. Like patient delay, a qualitative study provided insights into TB stigma experienced by women resulting in the concealment of diagnosis, expulsion from their community, or isolation; thus, delaying access to TB care and treatment[127]. Four qualitative studies mentioned self-perception of health, unconvinced diagnosis and need for TB treatment, and the perceived low effectiveness of TB treatment led to a delay in TB treatment initiation [48, 96, 119, 141]. We also found that retreatment cases were more likely to delay TB treatment initiation [94–98].

## Discussion

Our review is the first to focus on determinants of delayed TB diagnosis and treatment among high TB burden countries using evidence-based quantitative and qualitative information. Studies from high TB-burden LIC/LMIC reported longer median patient delay (28 days) than UMIC (10 days). Our findings were consistent with previous systematic reviews conducted in countries of different income levels [5, 8]. However, the median patient delay among UMIC in this review was shorter than the findings from observational studies conducted in other high-income countries [147, 148]. TB burden in high-income countries has been progressively reduced through improvements in socio-economic conditions, strong health systems components such as the delivery of TB services and universal health coverage, and social protection schemes [149]. Nevertheless, the high standards of living and wellbeing have shaped the notion that TB is not a significant concern, rendering a lower index of suspicion of TB and thus delaying TB care-seeking [150]. Notwithstanding, TB remains an issue, especially among hard-to-reach populations living in high-income settings like migrants [151, 152], who face challenges in accessing healthcare due to stigma, language barriers, and cost of testing and treatment [6].

The median health system delay in this review (LIC/LMIC: 14 days and UMIC: 4 days) was found to be shorter than previous systematic reviews conducted among countries of similar economies [5, 8]. As this review included studies conducted in the last decade, the improvement in health system delay may be attributed to the enhancements of healthcare systems [153] and the quality of TB laboratories [154]. The clinicians’ ability to consider TB as a differential diagnosis in high burden settings is also essential for early diagnosis and treatment [155]. However, there remains a paucity of data in several high TB burden countries, including seven in Africa (Central African Republic, Democratic Republic of Condo, Lesotho, Liberia, Namibia, Republic of the Congo, and Sierra Leone) and four in Asia (Democratic People’s Republic of Korea, Myanmar, Papua New Guinea, and Vietnam), potentially due to logistical challenges in conducting such studies.

### Patient delay

While TB is a disease mainly affecting men[156], in our review, we found that women faced challenges in accessing TB care promptly in some settings due to resource constraints, power imbalances, and poor TB knowledge. However, there was a paucity of sex-specific data on the determinants of delay in TB diagnosis and treatment. It is imperative to recognize sex disparities in TB care-seeking. Women with TB in high burden countries experienced delays in diagnosis and treatment because of barriers to TB services. Therefore, future studies should report disaggregated data by sex to inform programs and interventions addressing sex-specific vulnerabilities in improving access to TB services among men and women.

Despite wide coverage of free TB diagnostic and treatment services in high burden countries [157], people with TB and their families, especially the poor, bear the impact of high economic costs [158]. Studies included in our review also reported that livelihood, work, and family were prioritized and led to a delay in care-seeking. These factors, coupled with the physical environments and impoverished living conditions [127, 138], plunged low-income households into a vicious cycle of impoverishments [159, 160], making TB elimination overtly challenging. Aside from the broad expansion of TB services that have been shown to reduce the financial burden on TB-affected households [161], it is also essential to ensure that financial and social protection policies are in place to protect those at risk of catastrophic TB costs and poverty.

Our pooled estimates showed that rural dwellers were significantly associated with patient delay. In the rural setting, access to healthcare facilities, particularly an institution that offers TB diagnostic services, might be lacking [5]. Rural populations were also more likely to have a lower health literacy [162], resulting in poorer health status and outcomes [163]. Nevertheless, the concept of urban–rural is dynamic and context-dependent, driven by migration, population, and economic growth over time [164]. The consistent findings of rural residence and patient delay in TB [5, 8, 165] suggest increased efforts tailored to the country’s specific circumstances in reaching the affected communities are required.

In countries where TB diagnostic and treatment services are provided for free, access to TB care is further challenged by poor knowledge and awareness regarding such policies, making presumptive TB seek treatment early [123, 135]. In addition to poor awareness about the free TB treatment policy, we also identified studies that reported poor knowledge regarding TB symptoms associated with a delay in TB care-seeking. Therefore, people with TB would delay care-seeking until only when their illness compromised their ability to work and earn livelihoods [115]. Conventionally, symptomatic individuals are linked to TB transmission, and they are regarded as the target group for TB case-finding activities using the TB symptoms screening approach [166]. However, TB transmission could also occur during the subclinical (asymptomatic) phase, particularly heightened during episodes of symptoms exhibition unrelated to TB pathologies, such as bouts of either acute or chronic cough [167]. As people with subclinical TB might not report any symptoms, they have lower awareness and motivation to seek care; thus, leading to a delay in TB diagnosis and treatment and potentially sustaining TB transmission [168] in the household and community. Therefore, a better understanding of subclinical TB, its transmission dynamics, and the implications for TB control efforts are needed. Nevertheless, individuals who exhibit TB symptoms, such as cough, are more likely to have a higher bacillary load and transmit infection [169, 170]. Therefore, it is crucial to ensure that ill and symptomatic persons with TB are reached, tested, and treated promptly.

Furthermore, misperception regarding the causes of TB was also found to delay TB care-seeking. When no one in the family is ever diagnosed with TB, presumptive TB did not self-initiate care-seeking or is discouraged explicitly by family members to seek TB diagnosis and treatment [117]. Therefore, it is imperative first to measure the level of knowledge, awareness, and practices regarding TB in settings where studies as such have yet to be conducted. The gaps identified could then be used to develop health education programs and interventions about TB. Studies have shown that health education programs and dissemination of TB information effectively improve TB knowledge and awareness [171], enabling care-seeking and increasing the identification of TB cases [172]. Furthermore, understanding the knowledge and practices of health professionals could be done in parallel to improve care and facilitate the early identification of TB [173].

TB stigma continues to be a major barrier for people to access TB diagnosis and complete treatment [10, 143]. Moreover, stigma could also reduce the use of face masks [6, 174], further contributing to infection transmission. Despite being an increasingly important agenda of TB programs worldwide, there is a paucity of data on stigma [175], particularly information from the perpetrators of stigma [176]. There is also limited evidence on effective interventions that can reduce TB-related stigma [177]. Considering the importance of stigma reduction in TB control and elimination efforts, stigma should be systematically measured. De-stigmatisation must include approaches in healthcare institutions and beyond for a more inclusive care plan.

In high TB-burden countries, we found that people who presented with cough, fever, and night sweats were more likely to delay TB care-seeking. This is consistent with another systematic review conducted among low and middle-income countries [5]. The attribution of these symptoms to other respiratory infections or smoking and the inability to link them to TB was claimed as one of the primary reasons causing a delay in seeking care [138]. Contrarily, more severe symptoms such as hemoptysis were more likely to reduce delays in care-seeking. Therefore, education and awareness-raising activities could be recalibrated to specifically highlight the possibility of TB besides other respiratory illnesses in the event of more general symptoms such as cough and fever. Simultaneously, health workers' awareness on this matter in high-burden settings should be raised to improve TB case findings.

### Health system and treatment delay

Health system delay was more pronounced in LIC and LMIC than UMIC, likely due to the standard of health care, the strength of the national health systems, and the availability of resources. Among LIC and LMIC, a systematic review reported that the quality of health care in the public and private sectors was poor. The private sector relatively outperformed the public sector regarding the delivery of care and medicines availability [178]. The discrepancies in effectiveness and efficiency were highlighted as a facilitator to seek private healthcare, which eventually leads to a delay in TB diagnosis in a high TB burden setting like Cambodia[179]. Narrowing down to high TB-burden countries, the quality of public and private healthcare was also found to be below par, and systematic evaluations are needed to identify gaps in the TB care pathway [157].

Likewise, treatment delay was longer in LIC and LMIC than UMIC. The delay might be due to logistic factors such as long distance to treatment centers, availability of anti-TB drugs, and the absence of TB diagnostic services in local health facilities [96, 97]. Beyond systemic factors, individuals’ low perceived susceptibility and TB stigma could delay a person’s decision to initiate TB treatment [48, 127]. Interventions to decrease isolation post-diagnosis and social support should be provided to encourage prompt initiation of TB treatment [180]. Health providers also play a vital role in assisting people with TB to internalize the diagnosis and support them in decision-making [181].

### Strengths and limitations

To our knowledge, this systematic review will be the first to focus on countries with a high TB burden, where most of the TB cases in the world [2] are found. As the list consisted of countries from LIC, LMIC, and UMIC, we attempted to discern the differences in the determinants of delayed TB diagnosis and treatment between these countries.

However, we found high levels of heterogeneity amongst the studies potentially due to clinical and methodological diversities. We included studies from different high TB-burden countries and economic statuses. While we have restricted the study populations to people with presumptive TB and people with TB, their sociodemographic profiles were diverse. We acknowledged the limitation in analyzing data comprising of all possible subgroups in this review. Furthermore, we included the different observational non-randomized studies in this review. The design differed by temporality and the potential biases, contributing to methodological diversity.

In our attempt for comprehensiveness, we retained the threshold of delays as to how they were defined in individual studies. While it might not pose severe concerns for the narrative synthesis and pooling of median delays, the utilization of the delay threshold defined by individual studies in the meta-analysis of risk factors could lead to misinterpretation. Therefore, we pooled effect sizes from eligible studies that utilized similar delay thresholds in the meta-analyses. We incorporated heterogeneity into random-effects models using the Bayesian approach [23], which could yield more accurate interval estimates than conventional methods, especially for studies with a small sample size and are heterogenous [23, 182, 183]. However, the incorporation of heterogeneity in the random-effects models would not fully account for the clinical and methodological diversity in the studies. Analyses of study-level covariates in a meta-regression may be relevant to further investigate heterogeneities by the differences in studies characteristics and populations. We did not perform a meta-regression in this review, and this method could be considered in future reviews of similar nature.

Nevertheless, caution in interpreting and extrapolating the findings from the meta-analyses is warranted. For the pooled median delays, the overall estimates were influenced by studies in UMIC with larger sample sizes. Therefore, we opined that the pooled estimates by economies would be more informative. In the meta-analyses of risk factors, the pooling of estimates from studies with similar delay thresholds limited the number of studies that could be included. Most of the independent variables were also grouped differently, and we could not standardize them all. Hence, the meta-analyses were only performed for selected variables. However, we strived to maintain this review's comprehensiveness by triangulating findings from narrative synthesis and thematic analyses of qualitative studies.

This review did not include data from all 30 high TB-burden countries due to the absence of key outcome data and research activities. Notwithstanding the potential lack of representativeness due to the scarcity of data from several countries, this review highlights the gaps in knowledge and provides insights into the determinants of TB diagnosis and treatment delay in high-burden countries. However, the heterogeneity of the data limited the generalizability of our findings to settings underrepresented in this review.

## Conclusions

Our analyses revealed a substantial delay between the onset of TB symptoms and TB care-seeking among high burden countries, highlighting the need to continue to shape knowledge, change attitude, and raise awareness of the community, people at risk of TB, and the health providers. Specific vulnerabilities such as sex disparities in care-seeking, being older, and geographic isolation should be recognized and addressed through tailored approaches to improve access to TB services and early diagnosis [184]. It is also crucial to improve the consciousness of the society regarding TB to battle stigma, and networks [185] of support from within the families, the grassroots, and institutions could create an enabling environment for early care-seeking and treatment adherence and success. In contrast to patient delay, the shorter health system and treatment delay were encouraging. Nonetheless, TB programs should strive to test and treat TB by adopting WHO recommendations for same-day TB diagnosis [186] to further reduce TB transmission and mortality [187]. Higher-level policies and interventions such as health system strengthening, universal health coverage, and the provision of sustainable social welfare schemes are important to reduce delays, improve access to TB care, and ultimately achieve the global TB targets [188].

## Supplementary Information

**Additional file 1: Table S1.** Newcastle–Ottawa scale scores. **Table S2.** Critical Appraisal Skills Program (CASP) scores. **Table S3.** Computation of ConQual rating for patient delay. **Table S4.** Computation of ConQual score for health system delay. **Table S5.** Computation of ConQual score for treatment delay. Search strategy: EMBASE. Search strategy: PUBMED. Search strategy: CINAHL. Search strategy: PSYCINFO. **Figure S1.** Association between sex of individuals and health system delay.

## Data Availability

Not applicable. All data generated or analyzed during this study are included in this published article and its Additional file 1.

## Abbreviations

CASP: Critical Appraisal Skills Program
CI: Confidence interval
HIV: Human immunodeficiency virus
HQ: High quality
LIC: Low-income countries
LMIC: Lower-middle-income countries
LQ: Low quality
MQ: Medium quality
OR: Odds ratio
PRISMA: Preferred Reporting Items for Systematic Reviews and Meta-analysis
PROSPERO: International Prospective Register of Systematic Reviews
TB: Tuberculosis
UMIC: Upper-middle-income countries
USA: Unites States of America
WHO: World Health Organization
