# Supplementary material for: Duration and determinants of delayed tuberculosis diagnosis and treatment in high-burden countries: a mixed-methods systematic review and meta-analysis
Source: Respir Res. 2021 Sep 23;22:251. doi: 10.1186/s12931-021-01841-6 (PMC8459488; doi:10.1186/s12931-021-01841-6)
Supplement: Supplementary file 1 — Additional file 1: Table S1. Newcastle–Ottawa scale scores. Table S2. Critical Appraisal Skills Program (CASP) scores. Table S3. Computation of ConQual rating for patient delay. Table S4. Computation of ConQual score for health system delay. Table S5. Computation of ConQual score for treatment delay. Search strategy: EMBASE. Search strategy: PUBMED. Search strategy: CINAHL. Search strategy: PSYCINFO. Figure S1. Association between sex of individuals and health system delay. [file 12931_2021_1841_MOESM1_ESM.docx]

**Determinants of delayed diagnosis and treatment of tuberculosis in high-burden countries: a mixed-methods systematic review and meta-analysis**

**Additional Materials**

Contents

[Additional Table 1: Newcastle-Ottawa scale scores 2](#_Toc80618247)

[Additional table 2: Critical Appraisal Skills Program (CASP) scores 6](#_Toc80618248)

[Additional Table 3: Computation of ConQual rating for patient delay 8](#_Toc80618249)

[Additional Table 4: Computation of ConQual score for health system delay 10](#_Toc80618250)

[Additional Table 5: Computation of ConQual score for treatment delay 11](#_Toc80618251)

[Search strategy: EMBASE 12](#_Toc80618252)

[Search strategy: PUBMED 13](#_Toc80618253)

[Search strategy: CINAHL 15](#_Toc80618254)

[Search strategy: PSYCINFO 16](#_Toc80618255)

[Additional figure 1: Association between sex of individuals and health system delay 17](#_Toc80618256)

# **Additional Table 1: Newcastle-Ottawa scale scores**

| Case control studies^a^ | Country | Study design | Selection | | | | Comparability | Exposure | | | | Total score |
| --- | --- | --- | --- | --- | --- | --- | --- | --- | --- | --- | --- | --- |
|  |  |  | Representative cases | Case definition adequate | Selection of controls | Definition of controls | Confounding factors are controlled | Ascertainment of exposure | Same method of ascertainment for cases and controls | Non-response rate | |  |
| Coimbra 2012[107] | Brazil | CC | 1 | 1 | 0 | 1 | 1 | 1 | 1 | 1 | | 7 |
| Yirgu 2017[32] | Ethiopia | CC | 1 | 1 | 1 | 1 | 1 | 0 | 1 | 1 | | 7 |
| Cross-sectional studies^b^ | Country | Study design | Selection | | | | Comparability | | Outcome | | | Total score |
|  |  |  | Representative sample | Sample size | Non-respondents | Ascertainment of exposure | Confounding factors are controlled | Additional confounding factors are controlled | Assessment of the outcome | Statistical test | |  |
| Segagni Lusignani 2013[42] | Angola | CS | 1 | 1 | 0 | 1 | 1 | 1 | 1 | 1 | | 7 |
| Rifat 2011[43] | Bangladesh | CS | 1 | 1 | 0 | 1 | 1 | 0 | 1 | 1 | | 6 |
| Htike 2013[92] | Bangladesh | CS | 0 | 0 | 0 | 1 | 1 | 1 | 1 | 1 | | 5 |
| Maciel 2010[66] | Brazil | CS | 1 | 1 | 0 | 2 | 1 | 0 | 1 | 0 | | 6 |
| Machado 2011[65] | Brazil | CS | 1 | 0 | 0 | 1 | 1 | 1 | 1 | 1 | | 6 |
| Silva 2012[89] | Brazil | CS | 0 | 0 | 0 | 1 | 1 | 1 | 1 | 1 | | 5 |
| Maior 2012[64] | Brazil | CS | 1 | 0 | 0 | 1 | 0 | 0 | 1 | 1 | | 4 |
| Wysocki 2013[62] | Brazil | CS | 1 | 0 | 0 | 1 | 1 | 0 | 1 | 1 | | 5 |
| Deponti 2013[61] | Brazil | CS | 1 | 1 | 1 | 1 | 1 | 0 | 2 | 1 | | 8 |
| Trigueiro 2014[63] | Brazil | CS | 1 | 1 | 0 | 1 | 0 | 0 | 1 | 1 | | 5 |
| Almeida 2015[60] | Brazil | CS | 1 | 1 | 0 | 1 | 1 | 1 | 1 | 1 | | 7 |
| Lorent 2015[44] | Cambodia | MM^d^ | 1 | 0 | 0 | 1 | 1 | 0 | 1 | 1 | | 5 |
| Wang 2008[81] | China | CS | 1 | 1 | 0 | 1 | 0 | 0 | 1 | 1 | | 5 |
| Zhou 2012[68] | China | CS | 1 | 0 | 1 | 1 | 1 | 0 | 2 | 1 | | 7 |
| Li 2012[74] | China | CS | 1 | 1 | 0 | 1 | 1 | 0 | 1 | 1 | | 6 |
| Tobe 2013[73] | China | CS | 1 | 1 | 0 | 1 | 1 | 0 | 1 | 1 | | 6 |
| Duan 2013[72] | China | CS | 1 | 0 | 0 | 1 | 1 | 0 | 1 | 0 | | 4 |
| Zhao 2014[75] | China | CS | 1 | 0 | 0 | 1 | 1 | 0 | 2 | 1 | | 6 |
| Chen 2014[69] | China | CS | 1 | 1 | 0 | 1 | 1 | 1 | 1 | 1 | | 7 |
| Zhou 2014[98] | China | CS | 0 | 1 | 0 | 1 | 1 | 1 | 2 | 1 | | 7 |
| Lin 2015[80] | China | CS | 1 | 1 | 0 | 1 | 0 | 0 | 1 | 0 | | 4 |
| Xia 2016[76] | China | CS | 1 | 1 | 0 | 1 | 1 | 0 | 1 | 1 | | 6 |
| Chimbatata 2017[71] | China | CS | 1 | 0 | 0 | 1 | 1 | 0 | 2 | 1 | | 6 |
| Wang 2017[70] | China | CS | 1 | 1 | 0 | 1 | 1 | 1 | 1 | 1 | | 7 |
| Mesfin 2009[28] | Ethiopia | CS | 1 | 1 | 0 | 1 | 1 | 0 | 2 | 1 | | 7 |
| Yimer 2009[35] | Ethiopia | CS | 1 | 1 | 0 | 1 | 1 | 1 | 1 | 1 | | 7 |
| Gele 2009[31] | Ethiopia | CS | 0 | 0 | 0 | 1 | 1 | 1 | 1 | 1 | | 5 |
| Abebe 2010[33] | Ethiopia | CS | 1 | 1 | 0 | 1 | 1 | 1 | 1 | 1 | | 7 |
| Belay 2012[20] | Ethiopia | CS | 1 | 1 | 1 | 1 | 1 | 0 | 2 | 1 | | 8 |
| Hussen 2012[29] | Ethiopia | CS | 0 | 1 | 0 | 1 | 1 | 1 | 1 | 1 | | 6 |
| Senbeto 2013[34] | Ethiopia | CS | 1 | 1 | 0 | 1 | 1 | 1 | 1 | 1 | | 7 |
| Gebeyehu 2014[22] | Ethiopia | CS | 1 | 1 | 0 | 1 | 1 | 1 | 1 | 1 | | 7 |
| Addisu 2014[37] | Ethiopia | CS | 1 | 1 | 0 | 1 | 0 | 0 | 1 | 0 | | 4 |
| Yimer 2014[30] | Ethiopia | CS | 0 | 1 | 0 | 1 | 1 | 1 | 1 | 1 | | 6 |
| Asefa 2014[100] | Ethiopia | CS | 1 | 1 | 0 | 1 | 1 | 1 | 1 | 1 | | 7 |
| Engeda 2016[36] | Ethiopia | CS | 0 | 1 | 0 | 1 | 1 | 1 | 1 | 1 | | 6 |
| Gebreegziabher 2016[27] | Ethiopia | CS | 1 | 1 | 0 | 1 | 1 | 1 | 1 | 1 | | 7 |
| Bogale 2017[21] | Ethiopia | CS | 1 | 1 | 0 | 2 | 1 | 0 | 1 | 1 | | 7 |
| Adenager 2017[25] | Ethiopia | CS | 1 | 1 | 0 | 2 | 1 | 1 | 1 | 1 | | 8 |
| Asres 2017[26] | Ethiopia | CS | 1 | 1 | 0 | 1 | 1 | 1 | 1 | 1 | | 7 |
| Seid 2018[23] | Ethiopia | CS | 1 | 1 | 0 | 1 | 1 | 1 | 1 | 1 | | 7 |
| Fuge 2018[24] | Ethiopia | CS | 1 | 1 | 0 | 1 | 1 | 1 | 1 | 1 | | 7 |
| Satyanarayana 2012[50] | India | CS | 1 | 1 | 0 | 1 | 1 | 1 | 1 | 1 | | 7 |
| Tamhane 2012[47] | India | CS | 1 | 0 | 0 | 1 | 1 | 1 | 1 | 1 | | 6 |
| Thakur 2013[46] | India | CS | 1 | 1 | 0 | 1 | 1 | 1 | 1 | 1 | | 7 |
| Bronner Murrison 2016[101] | India | CS | 1 | 1 | 0 | 1 | 1 | 1 | 1 | 1 | | 7 |
| Purty 2016[45] | India | CS | 1 | 1 | 0 | 1 | 1 | 1 | 1 | 1 | | 7 |
| Basa 2016[48] | India | CS | 0 | 1 | 0 | 1 | 1 | 1 | 1 | 0 | | 5 |
| Kalra 2017[49] | India | CS | 1 | 1 | 0 | 1 | 1 | 0 | 1 | 1 | | 6 |
| Van Ness 2017[102] | India | CS | 1 | 1 | 0 | 1 | 1 | 1 | 1 | 1 | | 7 |
| Thomas 2018[93] | India | CS | 1 | 1 | 0 | 1 | 1 | 1 | 2 | 1 | | 8 |
| Mahendradhata 2008[103] | Indonesia | CS | 1 | 0 | 0 | 1 | 0 | 0 | 1 | 1 | | 4 |
| Lock 2011[51] | Indonesia | CS | 1 | 1 | 0 | 1 | 1 | 1 | 1 | 1 | | 7 |
| Ahmad 2013[52] | Indonesia | CS | 1 | 1 | 0 | 1 | 1 | 0 | 1 | 1 | | 6 |
| Ayuo 2008[53] | Kenya | CS | 0 | 0 | 0 | 1 | 0 | 0 | 1 | 1 | | 3 |
| Njau 2012[54] | Kenya | CS | 1 | 0 | 0 | 1 | 1 | 1 | 1 | 1 | | 6 |
| Saifodine 2013[38] | Mozambique | CS | 1 | 1 | 0 | 1 | 1 | 1 | 1 | 0 | | 6 |
| Fatiregun 2010[57] | Nigeria | CS | 1 | 0 | 0 | 1 | 1 | 0 | 2 | 0 | | 5 |
| Ukwaja 2013[56] | Nigeria | CS | 1 | 1 | 0 | 2 | 1 | 1 | 1 | 1 | | 8 |
| Biya 2014[55] | Nigeria | CS | 1 | 1 | 0 | 1 | 1 | 1 | 1 | 1 | | 7 |
| Adejumo 2016[88] | Nigeria | CS | 1 | 1 | 0 | 1 | 1 | 0 | 2 | 1 | | 7 |
| Bassili 2008[104] | Pakistan | CS | 1 | 1 | 0 | 1 | 1 | 1 | 1 | 1 | | 7 |
| Saqib 2011[105] | Pakistan | CS | 1 | 0 | 0 | 2 | 0 | 0 | 1 | 1 | | 5 |
| Saqib 2018[106] | Pakistan | CS | 1 | 1 | 0 | 0 | 1 | 1 | 1 | 1 | | 6 |
| Woith 2008[82] | Russia | CS | 1 | 0 | 1 | 2 | 0 | 0 | 2 | 1 | | 7 |
| Meintjes 2008[84] | South Africa | CS | 1 | 0 | 0 | 1 | 0 | 0 | 1 | 1 | | 4 |
| Van Wyk 2011[99] | South Africa | CS | 1 | 1 | 0 | 1 | 1 | 0 | 2 | 1 | | 7 |
| Moller 2011[83] | South Africa | CS | 1 | 1 | 0 | 1 | 0 | 0 | 0 | 1 | | 4 |
| Otwombe 2013[85] | South Africa | CS | 1 | 1 | 0 | 1 | 1 | 1 | 1 | 1 | | 7 |
| Mfinanga 2008[39] | Tanzania | CS | 1 | 1 | 0 | 1 | 1 | 1 | 1 | 1 | | 7 |
| Ngadaya 2009[40] | Tanzania | CS | 1 | 1 | 0 | 1 | 0 | 0 | 2 | 1 | | 6 |
| Hinderaker 2011[91] | Tanzania | CS | 1 | 1 | 0 | 1 | 1 | 1 | 1 | 1 | | 7 |
| Senkoro 2015[41] | Tanzania | CS | 1 | 1 | 0 | 1 | 1 | 1 | 1 | 1 | | 7 |
| Butsorn 2010[86] | Thailand | CS | 1 | 1 | 0 | 1 | 1 | 0 | 2 | 1 | | 7 |
| Rattananupong 2015[87] | Thailand | CS | 0 | 0 | 0 | 1 | 1 | 1 | 1 | 1 | | 5 |
| Chanda-Kapata 2016[58] | Zambia | CS | 1 | 1 | 0 | 1 | 1 | 1 | 1 | 1 | | 7 |
| Takarinda 2015[59] | Zimbabwe | CS | 1 | 1 | 0 | 1 | 1 | 1 | 1 | 1 | | 7 |
| Cohort studies^c^ | Country | Study design | Selection | | | | Comparability | | Outcome | | | Total score |
|  |  |  | Representative cases | Selection of non-exposed cohorts | Ascertainment of exposure | Outcome of interest was not present at start of study | Confounding factors are controlled | Additional confounding factors are controlled | Assessment of outcome | Follow-up long enough for outcome  to occur | Adequate  follow-up |  |
| Nogueira 2018[67] | Brazil | PC | 1 | 1 | 1 | 1 | 1 | 1 | 0 | 1 | 0 | 7 |
| Lin 2008[79] | China | RC | 1 | 1 | 1 | 1 | 1 | 1 | 1 | 1 | 0 | 8 |
| Xu 2013[78] | China | RC | 1 | 1 | 1 | 0 | 1 | 0 | 1 | 1 | 1 | 7 |
| Shu 2014[77] | China | PC | 1 | 1 | 1 | 1 | 1 | 0 | 1 | 0 | 0 | 6 |
| Paul 2012[94] | India | MM^e^ | 1 | 1 | 1 | 1 | 1 | 0 | 1 | 1 | 0 | 7 |
| Ilangovan 2015[96] | India | RC | 1 | 1 | 1 | 1 | 1 | 1 | 1 | 1 | 1 | 9 |
| Kant 2017[95] | India | RC | 1 | 0 | 1 | 1 | 1 | 1 | 1 | 1 | 0 | 7 |
| Boniface 2012[90] | South Africa | RC | 1 | 1 | 1 | 1 | 1 | 1 | 1 | 1 | 1 | 9 |
| Mugauri 2018[97] | Zimbabwe | RC | 1 | 1 | 1 | 1 | 1 | 1 | 1 | 1 | 1 | 9 |

CC, case control; CS, cross sectional; MM, mixed methods; RC, retrospective cohort; PC, prospective cohort

^a^The highest possible score for case-control studies was 9 (4 for selection, 2 for comparability, and 3 for exposure)

^b^The highest possible score for cross-sectional studies was 10 (5 for selection, 2 for comparability, and 3 for outcome)

^c^The highest possible score for cohort studies was 9 (4 for selection, 2 for comparability, and 3 for outcome)

^d^The observational study design (quantitative component) was cross sectional

^e^The observational study design (quantitative component) was retrospective cohort

# **Additional table 2: Critical Appraisal Skills Program (CASP) scores**

| First author and year | Country | Was there a clear statement of the aims of the research?^a^ | Is a qualitative methodology appropriate?^a^ | Was the research design appropriate to address the aims of the research?^a^ | Was the recruitment strategy appropriate to the aims of the research?^a^ | Was the data collected in a way that addressed the research issue?^a^ | Has the relationship between researcher and participants been adequately considered?^a^ | Have ethical issues been taken into considerations?^a^ | Was the data analysis sufficiently rigorous?^a^ | Is there a clear statement of findings?^a^ | How valuable is the research?^a^ | CASP score |
| --- | --- | --- | --- | --- | --- | --- | --- | --- | --- | --- | --- | --- |
| Bam 2014[114] | Bangladesh | 1 | 1 | 1 | 1 | 0.5 | 0 | 1 | 1 | 1 | 1 | 8.5 |
| Gosoniu 2008[113] | Bangladesh, India | 1 | 0.5 | 1 | 0.5 | 1 | 0 | 0.5 | 0.5 | 1 | 1 | 7 |
| Duarte de Sá 2013[127] | Brazil | 1 | 1 | 1 | 1 | 1 | 0.5 | 1 | 1 | 1 | 1 | 9.5 |
| Furlan 2014[128] | Brazil | 1 | 1 | 1 | 1 | 1 | 0 | 1 | 0.5 | 1 | 1 | 8.5 |
| Evangelista de Andrade 2016[129] | Brazil | 1 | 1 | 1 | 0.5 | 1 | 0.5 | 1 | 0.5 | 1 | 1 | 8.5 |
| Oliveira 2013[130] | Brazil | 1 | 1 | 0.5 | 0 | 0.5 | 0.5 | 1 | 0.5 | 1 | 1 | 7 |
| Lorent 2015[44] | Cambodia | 1 | 1 | 1 | 1 | 1 | 0.5 | 1 | 1 | 1 | 1 | 9.5 |
| Sundaram 2017[115] | Cambodia | 1 | 1 | 1 | 0.5 | 1 | 1 | 1 | 1 | 1 | 1 | 9.5 |
| Long 2008[133] | China | 1 | 1 | 1 | 1 | 1 | 0.5 | 1 | 1 | 1 | 1 | 9.5 |
| Wei 2009[132] | China | 1 | 1 | 1 | 1 | 1 | 0.5 | 1 | 1 | 1 | 1 | 9.5 |
| Hutchison 2017[134] | China | 1 | 1 | 1 | 1 | 1 | 1 | 1 | 1 | 1 | 1 | 10 |
| Strand 2011[131] | China | 1 | 0 | 1 | 1 | 0.5 | 0.5 | 0 | 0.5 | 1 | 1 | 6.5 |
| Gele 2010[110] | Ethiopia | 1 | 1 | 1 | 1 | 1 | 0.5 | 1 | 1 | 1 | 1 | 9.5 |
| Tadesse 2013[109] | Ethiopia | 1 | 1 | 1 | 1 | 1 | 0.5 | 1 | 1 | 1 | 1 | 9.5 |
| Sagbakken 2008[108] | Ethiopia | 1 | 1 | 1 | 1 | 1 | 0.5 | 1 | 1 | 1 | 1 | 9.5 |
| Mistry 2016[117] | India | 1 | 1 | 1 | 0.5 | 1 | 0 | 1 | 1 | 0 | 1 | 7.5 |
| Paul 2012[94] | India | 1 | 1 | 1 | 1 | 0.5 | 0 | 1 | 0.5 | 0 | 0.5 | 6.5 |
| Kapoor 2012[118] | India | 1 | 1 | 1 | 1 | 1 | 1 | 1 | 0.5 | 0 | 1 | 8.5 |
| Purohit 2015[116] | India | 1 | 1 | 1 | 1 | 1 | 0.5 | 1 | 1 | 1 | 1 | 9.5 |
| Yellappa 2017[120] | India | 1 | 1 | 1 | 1 | 1 | 0.5 | 1 | 1 | 1 | 1 | 9.5 |
| McArthur 2016[119] | India | 1 | 1 | 1 | 0.5 | 1 | 0.5 | 1 | 0.5 | 0.5 | 1 | 8 |
| Dewi 2016[122] | Indonesia | 1 | 1 | 1 | 1 | 1 | 0.5 | 1 | 0.5 | 1 | 1 | 9 |
| Rintiswati 2009[121] | Indonesia | 1 | 1 | 1 | 1 | 1 | 0.5 | 1 | 1 | 1 | 1 | 9.5 |
| Mindu 2017[111] | Mozambique | 1 | 1 | 1 | 1 | 1 | 0.5 | 1 | 1 | 1 | 1 | 9.5 |
| Oshi 2016[123] | Nigeria | 1 | 1 | 1 | 1 | 1 | 1 | 1 | 1 | 1 | 1 | 10 |
| Hu 2012[124] | Philippines | 1 | 1 | 1 | 1 | 1 | 1 | 1 | 1 | 1 | 1 | 10 |
| Kuznetsov 2013[135] | Russia | 1 | 1 | 1 | 1 | 1 | 1 | 1 | 1 | 1 | 1 | 10 |
| Kuznetsov 2014[136] | Russia | 1 | 1 | 1 | 1 | 1 | 0.5 | 1 | 1 | 1 | 1 | 9.5 |
| Adams 2017[137] | South Africa | 1 | 1 | 1 | 1 | 1 | 1 | 1 | 1 | 1 | 1 | 10 |
| Skordis-Worrall 2010[142] | South Africa | 1 | 1 | 1 | 1 | 1 | 0.5 | 1 | 1 | 1 | 1 | 9.5 |
| Murray 2013[141] | South Africa | 1 | 1 | 1 | 1 | 1 | 1 | 1 | 1 | 1 | 1 | 10 |
| Kerrigan 2017[140] | South Africa | 1 | 1 | 1 | 1 | 1 | 0.5 | 1 | 1 | 1 | 1 | 9.5 |
| Skinner 2016[139] | South Africa | 1 | 1 | 1 | 1 | 1 | 1 | 1 | 1 | 1 | 1 | 10 |
| Finnie 2011[138] | South Africa | 0.5 | 1 | 1 | 1 | 1 | 0.5 | 1 | 1 | 1 | 1 | 9 |
| Verhagen 2010[112] | Tanzania | 1 | 1 | 1 | 1 | 1 | 1 | 1 | 1 | 1 | 1 | 10 |
| Tschirhart 2016[143] | Thailand | 1 | 1 | 1 | 1 | 1 | 0.5 | 1 | 1 | 1 | 1 | 9.5 |
| Cremers 2016[125] | Zambia | 1 | 1 | 1 | 1 | 1 | 0.5 | 1 | 1 | 1 | 1 | 9.5 |
| Mavhu 2010[126] | Zimbabwe | 1 | 1 | 1 | 1 | 1 | 0.5 | 1 | 1 | 1 | 1 | 9.5 |

^a^CASP for qualitative study had 10 questions to critically appraise the paper. We gave a score of 1 if the paper fulfilled a criterion, 0.5 if we couldn’t tell if the paper fulfilled a criterion, and 0 if the paper did not fulfil a criterion

# **Additional Table 3: Computation of ConQual rating for patient delay**

| **Synthesized finding 1:** Perceived stigma and discrimination at workplace, within family and the community against women, and associating TB with HIV deterred presumptive TB cases from seeking TB diagnosis and care | | |
| --- | --- | --- |
| Dependability  Moderate  Long 2008[133], Duarte de Sá 2013[127], Furlan 2014[128], Hutchison 2017[134], Gosoniu 2008[113], Oshi 2016[123], Skinner 2016[139], McArthur 2016[119], Cremers 2016[125]  High  Adams 2017[137], Mavhu 2010[126], Skordis-Worrall 2010[142], Verhagen 2010[112], Hu 2012[124], Murray 2013[141], Kuznetsov 2013[135], Bam 2014[114], Kerrigan 2017[140], Sagbakken 2008[108], Finnie 2011[138], Sundaram 2017[115]  Dependability is high: level maintained as a majority of the individual findings have high level of dependability (12 high, 9 moderates) | Credibility  26 unequivocal findings  Credibility of findings is high | ConQual score  Confidence in the findings is high: high dependability and high credibility |
| **Synthesized finding 2:** Long distance to health facilities, and language barrier led to delay in care seeking and TB diagnosis | | |
| Dependability  Moderate  Tschirhart 2016[143], Hutchison 2017[134], Evangelista de Andrade 2016[129], Lorent 2015[44]  High  Gele 2010[110], Verhagen 2010[112], Hu 2012[124], Kuznetsov 2013[135], Kerrigan 2017[140], Tadesse 2013[109], Finnie 2011[138], Sundaram 2017[115]  Dependability is moderate: downgraded 1 level due to a  mixture of dependability among findings (4 high, 8 moderates) | Credibility  13 unequivocal findings  Credibility of findings is high | ConQual score  Confidence in the findings is moderate: downgraded 1 level due to moderate dependability and high credibility |
| **Synthesized finding 3:** Long chains of care seeking through multiple providers and the lack of trust in the health care system providing TB care led to delay in care seeking and TB diagnosis | | |
| Dependability  Moderate  Long 2008[133], Hutchison 2017[134], Gosoniu 2008[113], Oshi 2016[123], McArthur 2016[119], Evangelista de Andrade 2016[129], Cremers 2016[125], Wei 2009[132], Kapoor 2012[118]  High  Adams 2017[137], Gele 2010[110], Mindu 2017[111], Mavhu 2010[126], Skordis-Worrall 2010[142], Mistry 2016[117], Verhagen 2010[112], Hu 2012[124], Murray 2013[141], Kuznetsov 2013[135], Bam 2014[114], Kerrigan 2017[140], Dewi 2016[122], Rintiswati 2009[121], Tadesse 2013[109], Purohit 2015[116], Yellappa 2017[120], Sagbakken 2008[108], Finnie 2011[138], Sundaram 2017[115], Kuznetsov 2014[136]  Dependability is moderate: level maintained as a majority of the individual findings have high level of dependability (20 high, 9 moderates) | Credibility  36 unequivocal findings  Credibility of findings is high | ConQual score  Confidence in the findings is high: high dependability and high credibility |
| **Synthesized finding 3:** Gender-specific factors such as men dominating and owning the decision-making power in family, more economic constraints for women to seek healthcare, and men concealing health issues or denying disease severity by substance (alcohol and nicotine) abuse led to delay in care seeking and TB diagnosis | | |
| Dependability  Moderate  Tschirhart 2016[143], Duarte de Sá 2013[127], Gosoniu 2008[113], Oshi 2016[123]  High  Mavhu 2010[126], Skordis-Worrall 2010[142], Mistry 2016[117], Kuznetsov 2013[135], Bam 2014[114], Kerrigan 2017[140], Dewi 2016[122], Kuznetsov 2014[136]  Dependability is moderate: level maintained as a majority of the individual findings have high level of dependability (8 high, 4 moderates) | Credibility  14 unequivocal findings  Credibility of findings is high | ConQual score  Confidence in the findings is high: high dependability and high credibility |
| **Synthesized finding 4:** Competing priorities of livelihood, work, and family led to delay in care seeking and TB diagnosis | | |
| Dependability  Moderate  Tschirhart 2016[143], Wei 2009[132], Lorent 2015[44], Oshi 2016[123], Cremers 2016[125]  High  Hu 2012[124], Kuznetsov 2014[136]  Dependability is moderate: downgraded 1 level due to a  mixture of dependability among findings (2 high, 5 moderates) | Credibility  12 unequivocal findings  Credibility of findings is high | ConQual score  Confidence in the findings is moderate: downgraded 1 level due to moderate dependability and high credibility |
| **Synthesized finding 5:** Poor knowledge regarding TB symptoms and treatment, and the availability of free treatment policy were barriers to early healthcare seeking | | |
| Dependability  Moderate  Long 2008[133], Wei 2009[132], Duarte de Sá 2013[127], McArthur 2016[119], Cremers 2016[125]  High  Gele 2010[110], Mavhu 2010[126], Hu 2012[124], Kerrigan 2017[140], Rintiswati 2009[121], Sagbakken 2008[108], Finnie 2011[138], Kuznetsov 2014[136], Verhagen 2010[112]  Dependability is moderate: level maintained as a majority of the individual findings have high level of dependability (9 high, 5 moderates) | Credibility  17 unequivocal findings  Credibility of findings is high | ConQual score  Confidence in the findings is high: high dependability and high credibility |
| **Synthesized finding 6:** Presumptive TB cases delayed care-seeking due to low perceived severity of symptoms, low perceived susceptibility to TB, believed that TB is hereditary or retribution for sinful behavior, blame others for delay and then overpowered by hopelessness | | |
| Dependability  Moderate  Lorent 2015[44]  High  Mindu 2017[111], Hu 2012[124], Kuznetsov 2013[135], Dewi 2016[122], Sagbakken 2008[108], Sundaram 2017[115], Mistry 2016[117], Verhagen 2010[112], Bam 2014[114]  Dependability is moderate: level maintained as a majority of the individual findings have high level of dependability (9 high, 1 moderates) | Credibility  19 unequivocal findings  Credibility of findings is high | ConQual score  Confidence in the findings is high: high dependability and high credibility |

# **Additional Table 4: Computation of ConQual score for health system delay**

| **Synthesized finding 1:** Poor practice at the health facilities and ignorance of TB led to a delay in TB diagnosis | | |
| --- | --- | --- |
| Dependability  Moderate  Long 2008[133], Duarte de Sá 2013[127], Strand 2011[131]  High  Verhagen 2010[112], Bam 2014[114], Dewi 2016[122], Rintiswati 2009[121], Tadesse 2013[109], Yellappa 2017[120], Sagbakken 2008[108], Oliveira 2013[130], Purohit 2015[116]  Dependability is moderate: level maintained as a majority of the individual findings have high level of dependability (9 high, 3 moderates) | Credibility  19 unequivocal findings  Credibility of findings is high | ConQual score  Confidence in the findings is high: high dependability and high credibility |
| **Synthesized finding 2:** Complicated procedures at the health facilities to reach TB diagnosis | | |
| Dependability  Moderate  Furlan 2014[128], Paul 2012[94], Strand 2011[131]  High  Skordis-Worrall 2010[142], Mistry 2016[117], Hu 2012[124], Oliveira 2013[130]  Dependability is moderate: level maintained as a majority of the individual findings have high level of dependability (4 high, 3 moderates) | Credibility  8 unequivocal findings  Credibility of findings is high | ConQual score  Confidence in the findings is high: high dependability and high credibility |
| **Synthesized finding 3:** Lack of resources and materials in the health facilities led to a delay in TB diagnosis | | |
| Dependability  Moderate  Furlan 2014[128], Duarte de Sá 2013[127], Paul 2012[94]  High  Skordis-Worrall 2010[142], Oliveira 2013[130], Kuznetsov 2014[136]  Dependability is moderate: downgraded 1 level due to a  mixture of dependability among findings (2 high, 4 moderates) | Credibility  8 unequivocal findings  Credibility of findings is high | ConQual score  Confidence in the findings is moderate: downgraded 1 level due to moderate dependability and high credibility |

# **Additional Table 5: Computation of ConQual score for treatment delay**

| **Synthesized finding 1:** Self-perception of health and the unconvinced of the diagnosis and the effectiveness of TB treatment led to a delay in TB treatment initiation | | |
| --- | --- | --- |
| Dependability  Moderate  Skinner 2016[139], Paul 2012[94], Lorent 2015[44]  High  Mistry 2016[117]  Dependability is moderate: downgraded 1 level due to a  mixture of dependability among findings (1 high, 3 moderates) | Credibility  6 unequivocal findings  Credibility of findings is high | ConQual score  Confidence in the findings is moderate: downgraded 1 level due to moderate dependability and high credibility |
| **Synthesized finding 2:** Diagnosis and treatment initiated in different facilities caused a delay in TB treatment initiation | | |
| Dependability  High  Mistry 2016[117]  Dependability is high: Level maintained. Only 1 finding of high dependability | Credibility  1 unequivocal finding  Credibility of findings is high | ConQual score  Confidence in the findings is high: high dependability and high credibility |
| **Synthesized finding 3:** Geographical distance to health facilities and other competing priorities delayed TB treatment initiation | | |
| Dependability  Moderate  Skinner 2016[139]  Dependability is moderate: Level maintained. Only findings of moderate dependability | Credibility  2 unequivocal findings  Credibility of findings is high | ConQual score  Confidence in the findings is moderate: downgraded 1 level due to moderate dependability and high credibility |
| **Synthesized finding 4:** Health system factors such as lack of organization at the facilities to manage patients, poor staff attitude, and logistic issues caused a delay in TB treatment initiation | | |
| Dependability  Moderate  Skinner 2016[139], Paul 2012[94]  Dependability is moderate: Level maintained. Only findings of moderate dependability | Credibility  4 unequivocal findings  Credibility of findings is high | ConQual score  Confidence in the findings is moderate: downgraded 1 level due to moderate dependability and high credibility |
| **Synthesized finding 5:** Women experienced stigma due to TB diagnosis resulting in concealment of diagnosis or being isolated | | |
| Dependability  Moderate  Cremers 2016[125]  Dependability is moderate: Level maintained. Only 1 finding of moderate dependability) | Credibility  1 unequivocal finding  Credibility of findings is high | ConQual score  Confidence in the findings is moderate: downgraded 1 level due to moderate dependability and high credibility |

# **Search strategy: EMBASE**

('Lung tuberculosis'/exp OR tb':ti,ab,kw OR 'pulmonary tuberculosis':ti,ab,kw OR 'tuberculosis':ti,ab,kw OR 'mycobacterium tuberculosis':ti,ab,kw)

AND

('Delayed diagnosis'/exp OR 'health seeking behavior'/exp OR delay:ti,ab,kw OR delays:ti,ab,kw OR delayed:ti,ab,kw OR barrier:ti,ab,kw OR barriers:ti,ab,kw OR late:ti,ab,kw)

AND

(angola'/exp OR 'angolan'/exp OR 'bangladesh'/exp OR 'bangladeshi'/exp OR 'brazil'/exp OR 'brazilian'/exp OR 'cambodia'/exp OR 'cambodian'/exp OR 'central african republic'/exp OR 'central african'/exp OR 'china'/exp OR 'chinese'/exp OR 'congo'/exp OR 'congolese (kinshasa)'/exp OR 'congolese (brazzaville)'/exp OR 'north korea'/exp OR 'north korean'/exp OR 'ethiopia'/exp OR 'ethiopian'/exp OR 'india'/exp OR 'indian'/exp OR 'indonesia'/exp OR 'indonesian'/exp OR 'kenya'/exp OR 'kenyan'/exp OR 'lesotho'/exp OR 'sotho (people)'/exp OR 'liberia'/exp OR 'liberian'/exp OR 'mozambique'/exp OR 'mozambican'/exp OR 'myanmar'/exp OR 'burmese'/exp OR 'namibia'/exp OR 'namibian'/exp OR 'nigeria'/exp OR 'nigerian'/exp OR 'pakistan'/exp OR 'pakistani'/exp OR 'papua new guinea'/exp OR 'papua new guinean'/exp OR 'philippines'/exp OR 'filipino (people)'/exp OR 'russian federation'/exp OR 'russian (people)'/exp OR 'russian (citizen)'/exp OR 'sierra leone'/exp OR 'sierra leonean'/exp OR 'south africa'/exp OR 'south african'/exp OR 'tanzania'/exp OR 'tanzanian'/exp OR 'thailand'/exp OR 'thai (people)'/exp OR 'viet nam'/exp OR 'vietnamese'/exp OR 'zambia'/exp OR 'zambian'/exp OR 'zimbabwe'/exp OR 'zimbabwean'/exp OR 'angola':ti,ab,kw OR 'angolan':ti,ab,kw OR 'bangladesh':ti,ab,kw OR 'bangladeshi':ti,ab,kw OR 'brazil':ti,ab,kw OR 'brazilian':ti,ab,kw OR 'cambodia':ti,ab,kw OR 'cambodian':ti,ab,kw OR 'central african republic':ti,ab,kw OR 'central african':ti,ab,kw OR 'china':ti,ab,kw OR 'chinese':ti,ab,kw OR 'congo':ti,ab,kw OR 'congolese (kinshasa)':ti,ab,kw OR 'congolese (brazzaville)':ti,ab,kw OR 'north korea':ti,ab,kw OR 'north korean':ti,ab,kw OR 'ethiopia':ti,ab,kw OR 'ethiopian':ti,ab,kw OR 'india':ti,ab,kw OR 'indian':ti,ab,kw OR 'indonesia':ti,ab,kw OR 'indonesian':ti,ab,kw OR 'kenya':ti,ab,kw OR 'kenyan':ti,ab,kw OR 'lesotho':ti,ab,kw OR 'sotho (people)':ti,ab,kw OR 'liberia':ti,ab,kw OR 'liberian':ti,ab,kw OR 'mozambique':ti,ab,kw OR 'mozambican':ti,ab,kw OR 'myanmar':ti,ab,kw OR 'burmese':ti,ab,kw OR 'namibia':ti,ab,kw OR 'namibian':ti,ab,kw OR 'nigeria':ti,ab,kw OR 'nigerian':ti,ab,kw OR 'pakistan':ti,ab,kw OR 'pakistani':ti,ab,kw OR 'papua new guinea':ti,ab,kw OR 'papua new guinean':ti,ab,kw OR 'philippines':ti,ab,kw OR 'filipino (people)':ti,ab,kw OR 'russian federation':ti,ab,kw OR 'russian (people)':ti,ab,kw OR 'russian (citizen)':ti,ab,kw OR 'sierra leone':ti,ab,kw OR 'sierra leonean':ti,ab,kw OR 'south africa':ti,ab,kw OR 'south african':ti,ab,kw OR 'tanzania':ti,ab,kw OR 'tanzanian':ti,ab,kw OR 'thailand':ti,ab,kw OR 'thai (people)':ti,ab,kw OR 'viet nam':ti,ab,kw OR 'vietnamese':ti,ab,kw OR 'zambia':ti,ab,kw OR 'zambian':ti,ab,kw OR 'zimbabwe':ti,ab,kw OR 'zimbabwean':ti,ab,kw)

# **Search strategy: PUBMED**

"Tuberculosis, Pulmonary"[Mesh] OR tb[Title/Abstract] OR pulmonary tuberculosis[Title/Abstract] OR tuberculosis[Title/Abstract] OR mycobacterium tuberculosis[Title/Abstract] OR tb[Text Word] OR pulmonary tuberculosis[Text Word] OR tuberculosis[Text Word] OR mycobacterium tuberculosis[Text Word] OR tb[Other Term] OR pulmonary tuberculosis[Other Term] OR tuberculosis[Other Term] OR mycobacterium tuberculosis[Other Term]

AND

"Delayed Diagnosis"[Mesh] OR "Patient Acceptance of Health Care"[Mesh] OR Delay [Title/Abstract] OR delays [Title/Abstract] OR delayed[Title/Abstract] OR barrier[Title/Abstract] OR barriers[Title/Abstract] OR late[Title/Abstract] OR Delay[Text Word] OR delays[Text Word] OR delayed[Text Word] OR barrier[Text Word] OR barriers[Text Word] OR late[Text Word] OR Delay[Other Term] OR delays[Other Term] OR delayed[Other Term] OR barrier[Other Term] OR barriers[Other Term] OR late[Other Term] OR health seek*[Title/Abstract] OR health seek* behavio*[Title/Abstract] OR health seek*[Text Word] OR health seek* behavio*[Text Word] OR health seek*[Other Term] OR health seek* behavio*[Other Term]

AND

"Angola"[Mesh] OR "Bangladesh"[Mesh] OR "Brazil"[Mesh] OR "Cambodia"[Mesh] OR "Central African Republic"[Mesh] OR "China"[Mesh] OR "Congo"[Mesh] OR "Democratic Republic of the Congo"[Mesh] OR "Democratic People's Republic of Korea"[Mesh] OR "Ethiopia"[Mesh] OR "India"[Mesh] OR "Indonesia"[Mesh] OR "Kenya"[Mesh] OR "Lesotho"[Mesh] OR "Liberia"[Mesh] OR "Mozambique"[Mesh] OR "Myanmar"[Mesh] OR "Namibia"[Mesh] OR "Nigeria"[Mesh] OR "Pakistan"[Mesh] OR "Papua New Guinea"[Mesh] OR "Philippines"[Mesh] OR "Russia"[Mesh] OR "Sierra Leone"[Mesh] OR "South Africa"[Mesh] OR "Tanzania"[Mesh] OR "Thailand"[Mesh] OR "Vietnam"[Mesh] OR "Zambia"[Mesh] OR "Zimbabwe"[Mesh] OR "angola"[Title/Abstract]) OR "angolan"[Title/Abstract]) OR "bangladesh"[Title/Abstract]) OR "bangladeshi"[Title/Abstract]) OR "brazil"[Title/Abstract]) OR "brazilian"[Title/Abstract]) OR "cambodia"[Title/Abstract]) OR "cambodian"[Title/Abstract]) OR "central africa republic"[Title/Abstract]) OR "central african"[Title/Abstract]) OR "china"[Title/Abstract]) OR "chinese"[Title/Abstract]) OR "congo"[Title/Abstract]) OR "democratic republic of congo"[Title/Abstract]) OR "kinshasa"[Title/Abstract]) OR "brazzaville"[Title/Abstract]) OR "congolese"[Title/Abstract]) OR "north korea"[Title/Abstract]) OR "north korean"[Title/Abstract]) OR "ethiopia"[Title/Abstract]) OR "ethiopian"[Title/Abstract]) OR "india"[Title/Abstract]) OR "indian"[Title/Abstract]) OR "indonesia"[Title/Abstract]) OR "indonesian"[Title/Abstract]) OR "kenya"[Title/Abstract]) OR "kenyan"[Title/Abstract]) OR "lesotho"[Title/Abstract]) OR "sotho"[Title/Abstract]) OR "liberia"[Title/Abstract]) OR "liberian"[Title/Abstract]) OR "mozambique"[Title/Abstract]) OR "mozambican"[Title/Abstract]) OR "myanmar"[Title/Abstract]) OR "burmese"[Title/Abstract]) OR "burma"[Title/Abstract]) OR "namibia"[Title/Abstract]) OR "namibian"[Title/Abstract]) OR "nigeria"[Title/Abstract]) OR "nigerian"[Title/Abstract]) OR "pakistan"[Title/Abstract]) OR "pakistani"[Title/Abstract]) OR "papua new guinea"[Title/Abstract]) OR "papua new guinean"[Title/Abstract]) OR "philippines"[Title/Abstract]) OR "filipino"[Title/Abstract]) OR "russia"[Title/Abstract]) OR "russian"[Title/Abstract]) OR "sierra leone"[Title/Abstract]) OR "sierra leonean"[Title/Abstract]) OR "south africa"[Title/Abstract]) OR "south african"[Title/Abstract]) OR "tanzania"[Title/Abstract]) OR "tanzanian"[Title/Abstract]) OR "thailand"[Title/Abstract]) OR "thai"[Title/Abstract]) OR "vietnam"[Title/Abstract]) OR "vietnamese"[Title/Abstract]) OR "zambia"[Title/Abstract]) OR "zambian"[Title/Abstract]) OR "zimbabwe"[Title/Abstract]) OR "zimbabwean"[Title/Abstract] OR "angola"[Text Word] OR "angolan"[Text Word] OR "bangladesh"[Text Word] OR "bangladeshi"[Text Word] OR "brazil"[Text Word] OR "brazilian"[Text Word] OR "cambodia"[Text Word] OR "cambodian"[Text Word] OR "central africa republic"[Text Word] OR "central african"[Text Word] OR "china"[Text Word] OR "chinese"[Text Word] OR "congo"[Text Word] OR "democratic republic of congo"[Text Word] OR "kinshasa"[Text Word] OR "brazzaville"[Text Word] OR "congolese"[Text Word] OR "north korea"[Text Word] OR "north korean"[Text Word] OR "ethiopia"[Text Word] OR "ethiopian"[Text Word] OR "india"[Text Word] OR "indian"[Text Word] OR "indonesia"[Text Word] OR "indonesian"[Text Word] OR "kenya"[Text Word] OR "kenyan"[Text Word] OR "lesotho"[Text Word] OR "sotho"[Text Word] OR "liberia"[Text Word] OR "liberian"[Text Word] OR "mozambique"[Text Word] OR "mozambican"[Text Word] OR "myanmar"[Text Word] OR "burmese"[Text Word] OR "burma"[Text Word] OR "namibia"[Text Word] OR "namibian"[Text Word] OR "nigeria"[Text Word] OR "nigerian"[Text Word] OR "pakistan"[Text Word] OR "pakistani"[Text Word] OR "papua new guinea"[Text Word] OR "papua new guinean"[Text Word] OR "philippines"[Text Word] OR "filipino"[Text Word] OR "russia"[Text Word] OR "russian"[Text Word] OR "sierra leone"[Text Word] OR "sierra leonean"[Text Word] OR "south africa"[Text Word] OR "south african"[Text Word] OR "tanzania"[Text Word] OR "tanzanian"[Text Word] OR "thailand"[Text Word] OR "thai"[Text Word] OR "vietnam"[Text Word] OR "vietnamese"[Text Word] OR "zambia"[Text Word] OR "zambian"[Text Word] OR "zimbabwe"[Text Word] OR "zimbabwean"[Text Word]

# **Search strategy: CINAHL**

(MH "Tuberculosis, Pulmonary") OR TI TB OR AB TB OR MM TB OR TI tuberculosis OR AB tuberculosis OR MM tuberculosis OR TI pulmonary tuberculosis OR AB pulmonary tuberculosis OR MM pulmonary tuberculosis OR TI mycobacterium tuberculosis OR AB mycobacterium tuberculosis OR MM mycobacterium tuberculosis

AND

(MH "Health Seeking Behavior (Iowa NOC)") OR (MH "Health Seeking Behaviors (NANDA)") OR (MH "Diagnosis, Delayed") OR TI delay OR AB delay OR MM delay OR TI delays OR AB delays OR MM delays OR TI delayed OR AB delayed OR MM delayed OR TI barrier OR AB barrier OR MM barrier OR TI barriers OR AB barriers OR MM barriers OR TI late OR AB late OR MM late

Final combination: Filtered by Geography: Asia, Europe, Africa, Mexico/Central and South America

# **Search strategy: PSYCINFO**

MeSH and key workds: (exp Pulmonary Tuberculosis/ or pulmonary tuberculosis.mp.) OR Title: (tb or pulmonary tuberculosis or tuberculosis or mycobacterium tuberculosis) OR Abstract: (tb or pulmonary tuberculosis or tuberculosis or mycobacterium tuberculosis) OR Key concepts: (tb or pulmonary tuberculosis or tuberculosis or mycobacterium tuberculosis)

AND

MeSH and key words: (exp Health Care Seeking Behavior/ or health seeking.mp.) OR MeSH and key words: (exp Diagnosis/ or delayed diagnosis.mp.) OR Title (delay or delays or delayed or barrier or barriers or late) OR Abstract: (delay or delays or delayed or barrier or barriers or late) OR Key concepts: (delay or delays or delayed or barrier or barriers or late)

AND

Title: (angola or angolan or bangladesh or bangladeshi or brazil or brazilian or cambodia or cambodian or central african republic or central african or china or chinese or congo or congolese or kinshasa or brazzaville or north korea or north korean or ethiopia or ethiopian or india or indian or indonesia or indonesian or kenya or kenyan or lesotho or sotho or liberia or liberian or mozambique or mozambican or myanmar or burmese or namibia or namibian or nigeria or nigerian or pakistan or pakistani or papua new guinea or papua new guinean or philippines or filipino or russian federation or russia or russian or sierra leone or sierra leonean or south africa or south african or tanzania or tanzanian or thailand or thai or vietnam or vietnamese or zambia or zambian or zimbabwe or zimbabwean) OR Abstract: (angola or angolan or bangladesh or bangladeshi or brazil or brazilian or cambodia or cambodian or central african republic or central african or china or chinese or congo or congolese or kinshasa or brazzaville or north korea or north korean or ethiopia or ethiopian or india or indian or indonesia or indonesian or kenya or kenyan or lesotho or sotho or liberia or liberian or mozambique or mozambican or myanmar or burmese or namibia or namibian or nigeria or nigerian or pakistan or pakistani or papua new guinea or papua new guinean or philippines or filipino or russian federation or russia or russian or sierra leone or sierra leonean or south africa or south african or tanzania or tanzanian or thailand or thai or vietnam or vietnamese or zambia or zambian or zimbabwe or zimbabwean) OR Key concepts: (angola or angolan or bangladesh or bangladeshi or brazil or brazilian or cambodia or cambodian or central african republic or central african or china or chinese or congo or congolese or kinshasa or brazzaville or north korea or north korean or ethiopia or ethiopian or india or indian or indonesia or indonesian or kenya or kenyan or lesotho or sotho or liberia or liberian or mozambique or mozambican or myanmar or burmese or namibia or namibian or nigeria or nigerian or pakistan or pakistani or papua new guinea or papua new guinean or philippines or filipino or russian federation or russia or russian or sierra leone or sierra leonean or south africa or south african or tanzania or tanzanian or thailand or thai or vietnam or vietnamese or zambia or zambian or zimbabwe or zimbabwean) OR Location: (angola or angolan or bangladesh or bangladeshi or brazil or brazilian or cambodia or cambodian or central african republic or central african or china or chinese or congo or congolese or kinshasa or brazzaville or north korea or north korean or ethiopia or ethiopian or india or indian or indonesia or indonesian or kenya or kenyan or lesotho or sotho or liberia or liberian or mozambique or mozambican or myanmar or burmese or namibia or namibian or nigeria or nigerian or pakistan or pakistani or papua new guinea or papua new guinean or philippines or filipino or russian federation or russia or russian or sierra leone or sierra leonean or south africa or south african or tanzania or tanzanian or thailand or thai or vietnam or vietnamese or zambia or zambian or zimbabwe or zimbabwean)

# **Additional figure 1: Association between sex of individuals and health system delay**

| 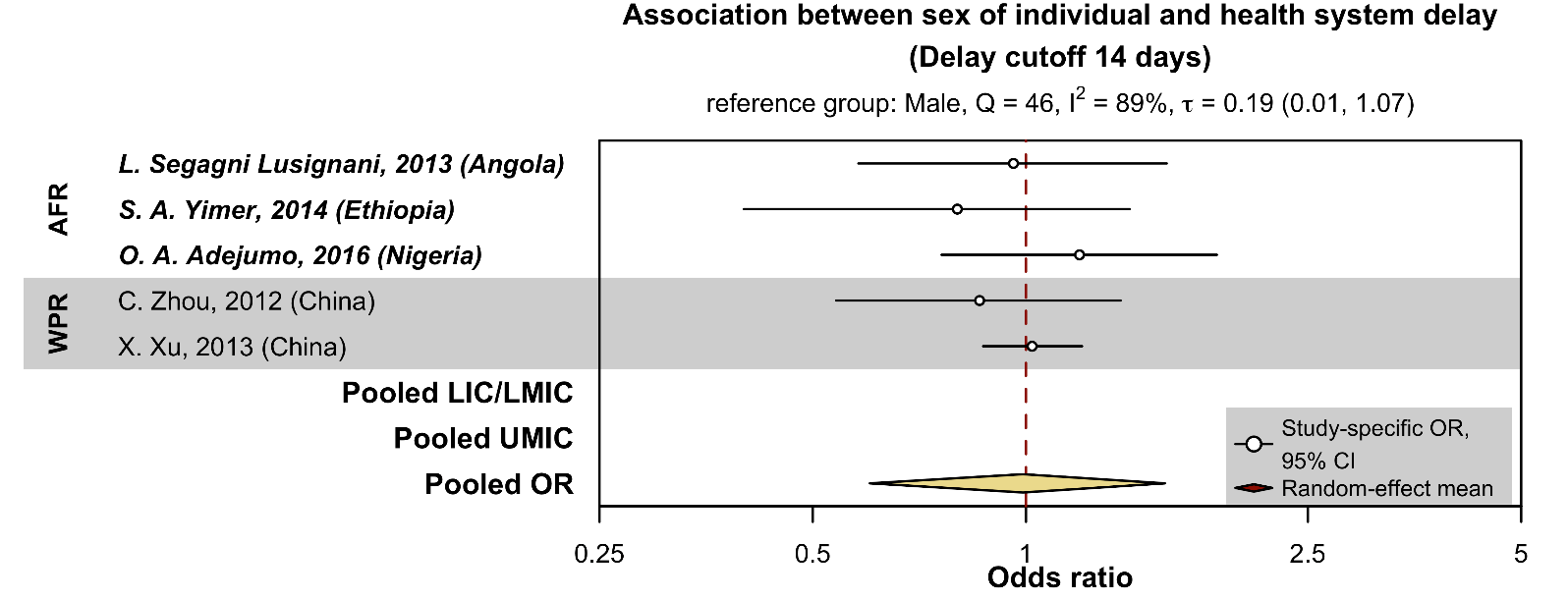 |
| --- |
| **Additional figure 1: ﻿Association between sex of individuals and health system delay.** Countries were grouped by WHO region (AFR; African Region, AMR; Region of the Americas, SEAR; South-East Asia Region, WPR; Western Pacific Region) and categorized as (i) LIC/LMIC (low- or lower-middle-income countries), or (ii) UMIC (upper-middle-income countries) as designated by the World Bank in 2019. The reference group for sex was male. The odds ratio (OR) were pooled (in yellow) by countries’ economic status using Bayesian random-effects meta-analysis. Odds ratios are presented in the log scale. |
